# Selecting Optimal Random Forest Predictive Models: A Case Study on Predicting the Spatial Distribution of Seabed Hardness

**DOI:** 10.1371/journal.pone.0149089

**Published:** 2016-02-18

**Authors:** Jin Li, Maggie Tran, Justy Siwabessy

**Affiliations:** Geoscience Australia, GPO Box 378, Canberra, ACT, 2601, Australia; Universitat Rovira i Virgili, SPAIN

## Abstract

Spatially continuous predictions of seabed hardness are important baseline environmental information for sustainable management of Australia’s marine jurisdiction. Seabed hardness is often inferred from multibeam backscatter data with unknown accuracy and can be inferred from underwater video footage at limited locations. In this study, we classified the seabed into four classes based on two new seabed hardness classification schemes (i.e., hard90 and hard70). We developed optimal predictive models to predict seabed hardness using random forest (RF) based on the point data of hardness classes and spatially continuous multibeam data. Five feature selection (FS) methods that are variable importance (VI), averaged variable importance (AVI), knowledge informed AVI (KIAVI), Boruta and regularized RF (RRF) were tested based on predictive accuracy. Effects of highly correlated, important and unimportant predictors on the accuracy of RF predictive models were examined. Finally, spatial predictions generated using the most accurate models were visually examined and analysed. This study confirmed that: 1) hard90 and hard70 are effective seabed hardness classification schemes; 2) seabed hardness of four classes can be predicted with a high degree of accuracy; 3) the typical approach used to pre-select predictive variables by excluding highly correlated variables needs to be re-examined; 4) the identification of the important and unimportant predictors provides useful guidelines for further improving predictive models; 5) FS methods select the most accurate predictive model(s) instead of the most parsimonious ones, and AVI and Boruta are recommended for future studies; and 6) RF is an effective modelling method with high predictive accuracy for multi-level categorical data and can be applied to ‘small *p* and large *n*’ problems in environmental sciences. Additionally, automated computational programs for AVI need to be developed to increase its computational efficiency and caution should be taken when applying filter FS methods in selecting predictive models.

## Introduction

Seabed substrate data is important baseline environmental information for supporting the sustainable management of Australia’s marine jurisdiction. Seabed substrate is an important factor controlling the spatial distribution of benthic marine communities as it influences the colonisation and formation of ecological communities and the abundance of benthic organisms [1–6]. Seabed hardness is an important character of seabed substrate as it may influence the nature of attachment of an organism to the seabed [6]. Hard substrates provide environments that generally support sessile suspension feeders, while soft (unconsolidated) substrates generally support discrete motile invertebrates [5]. Hence, a spatially continuous measurement of seabed hardness would be a significant aid in predicting the spatial distribution of benthic marine communities and thus to marine ecosystem management. Moreover, it can also be used for the sustainable exploitation of marine resources and planning infrastructure (e.g. selection of pipeline routes).

Despite its importance, seabed hardness data is often difficult to acquire [7]. It can be directly measured at (often widely-dispersed) discrete locations, inferred from underwater video footage at discrete locations over small areas [8], or inferred from multibeam backscatter data [9–11]. However, there are disadvantages associated with these methods. For example, the direct measurements are only available at point locations, and the inferred data are either only available at discrete locations over small areas or their accuracy is unknown and may be affected by many factors [7, 12]. Therefore, predictive modelling provides an alternative approach to generate spatially continuous data of seabed hardness [7], where seabed hardness was classified into two classes (i.e. hard/soft binary data) according to the approach by Stein *et al*. [8] that is however difficult to use for certain substratum compositions. Moreover, all mixed classes were classified into hard class [7], so the relevant information of the mixed classes is missing for the management of associated habitats. However, no study has been conducted on predicting seabed hardness based on four classes data yet.

Predictive variables are essential to making predictions of seabed hardness. Physical properties derived from multibeam backscatter and bathymetry have proven to be useful predictors for predicting seabed hardness [7, 12]. Multibeam data collection can result in hardness and roughness maps which differentiate between different substrate types of the seabed [13]. At regional and continental scales, seabed sediments are often strongly correlated to bathymetry [14, 15]. At these scales, bathymetry and its derived properties are informative predictors of seabed substrate types. Moreover, recent technological advancements in sonar equipment have significantly increased the amount of biophysical data for making spatially continuous predictions of seabed hardness.

To predict seabed hardness, one of the most important decisions to make is to identify the best modelling technique that can generate a surface to truthfully represent seabed hardness. Random forest (RF) developed by Ho [16, 17] and Breiman [18, 19] has proven to have high predictive accuracy (PA) in data mining and many other disciplines [20–24]. It outperformed a number of statistical modelling techniques for spatial prediction using continuous data in the marine environmental sciences [14, 25, 26]. Hence, it was applied to predicting the spatial distribution of seabed hardness based on two classes and again achieved high PA [7]. In a recent study, seabed substrate types were predicted based on four textural classes derived from relative proportions of sediment grain size and their ratio [27]; and RF was again found to be one of the most accurate methods. However, its predictions were for grain size ranging from mud to gravel, which falls into the soft class according to Li *et al*. [7]. In addition, a Self-Organising Map and a hierarchical clustering method were jointly applied to angular backscatter response curves and produced seabed hardness classification with multiple classes, but its accuracy was less than RF and the results were not spatially continuous [12].

Model selection is essential for identifying an optimal predictive model and various methods have been developed [28–30]. However, it is often argued that model selection is less important for RF, because: 1) RF is insensitive to un-important variables, as it selects the most important variable at each node split [23]; 2) it is of high predictive performance even when most predictive variables are noisy [21]; and 3) its PA depends only on the number of strong features and not on the number of noisy variables if sample sizes are large (500 to 1000) [31]. It was found that excluding the correlated variables may improve the PA [25, 32, 33]. In contrast, it was observed that including some correlated variables could improve the PA [7, 15, 34, 35], suggesting that correlated variables may be able to compensate for the small number of predictors generally found in the environmental sciences. These contradictory findings demonstrate that model selection is necessary for identifying an optimal predictive model for RF.

A model selection procedure for RF was developed previously by Li *et al*. [7] and it selects the predictors based on the variable importance produced from RF [36]. This is a stepwise procedure using both forward and backward selection to add or eliminate predictors, which is similar to what has been proposed in recent studies [37, 38], but uses PA to determine the selection of each predictive variable. In the environmental sciences, predictive variables are often correlated, which may affect the observed variable importance for the predictors when using RF. To deal with this, an R package ‘*extendedForest*’ [39] was developed to compensate for the shortcomings in the existing RF package by Liaw and Wiener [36]. Furthermore, two R functions, Boruta [40] and RRF (i.e. regularized RF) [41], were developed to automatically search for the important predictors for RF. All these studies provide fundamental tools for selecting the important predictors in this study.

In this study, we aim to select the most accurate model to predict the spatial distribution of seabed hardness based on four classes of seabed hardness. To achieve this, we: 1) introduced two new classification schemes for seabed video classification; 2) tested the effects of various predictor sets on the accuracy of RF predictive models based on video classifications and seabed biophysical variables; and 3) examined the influence of five feature selection (FS) methods on the most accurate predictive model identified. Finally, the most accurate models were used to predict the spatial distribution of seabed hardness and the predictions were visually examined and compared with the predictions of hardness in two classes [7].

## Methods

### Study region

**Fig 1:**
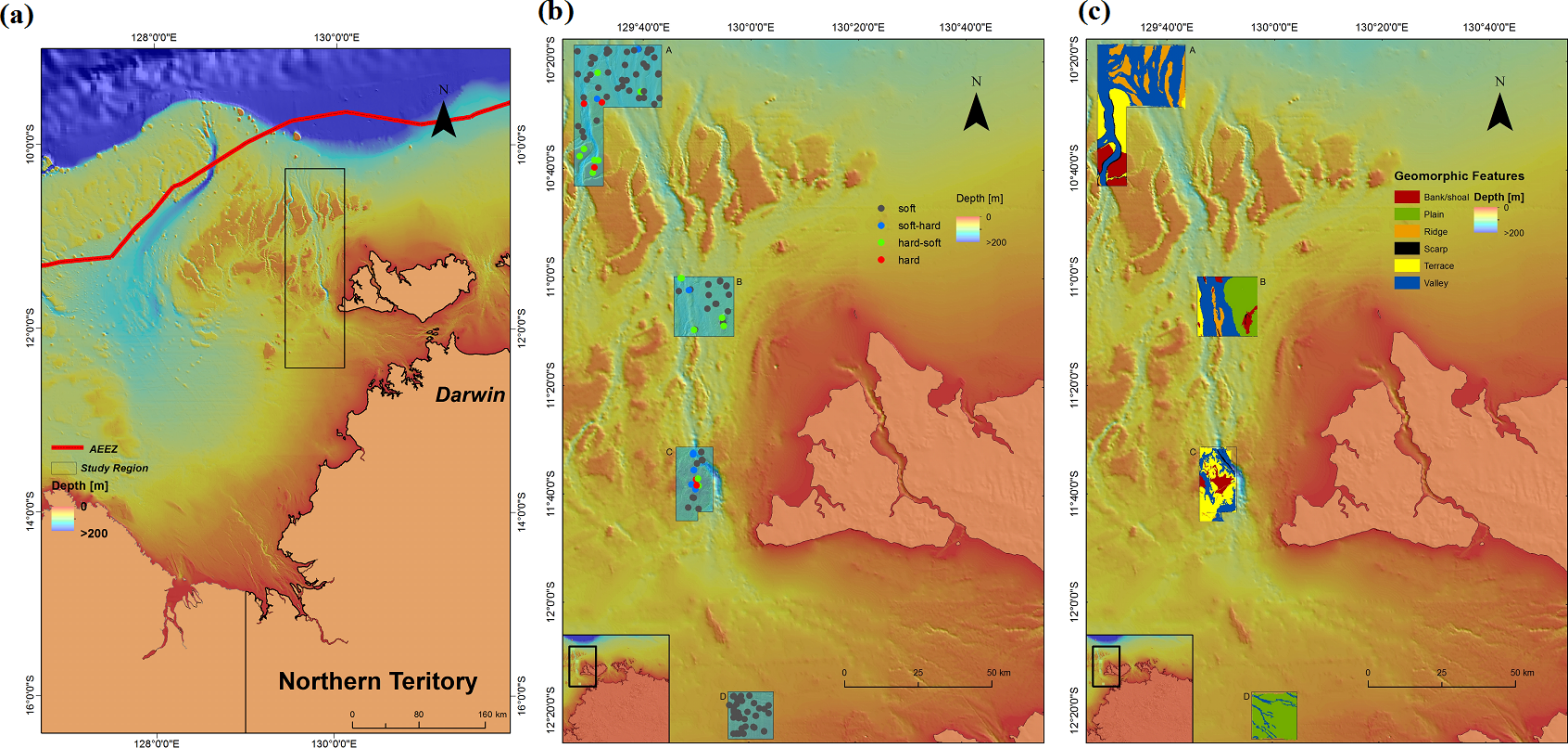
a) Location of the study region in the eastern Joseph Bonaparte Gulf, northern Australian marine margin overlaid with bathymetry; b) location of the four study areas (A, B, C, and D) in the study region and seabed hardness types (hard, hard-soft, soft-hard and soft) based on hard90 overlaid with bathymetry at video transect; and c) the geomorphic features of the four study areas.

The study region is located in the eastern Joseph Bonaparte Gulf, northern Australian marine margin (Fig 1A). Four areas (A—D) in the region were used in this study (Fig 1B), which were surveyed in 2009 [42] and 2010 [43] under the permissions of Geoscience Australia and Department of the Environment, Water Heritage and the Arts. In these surveys, high-resolution multibeam bathymetry and backscatter data and co-located underwater video transects were acquired across the four areas (Fig 1B). The areas comprise a spatially complex suite of geomorphic features including shallow flat-topped banks, terraces, ridges, deep valleys and plains (Fig 1C).

### Schemes for deriving seabed hardness from underwater video observations

#### Estimation of substratum composition

The seabed and associated epibenthos (Fig 1B) were recorded along underwater video transects using a forward-facing towed-video system. The video footage was analysed based on a 15-second window for each transect to classify substratum composition [44]. The substratum composition was visually estimated to 5% precision [45] in terms of seven size-class categories of rock, boulders, cobble, rubble, gravel, sand and mud as defined by [46]. While this method is subjective and may not be as precise as the regularly used point-count method, previous studies have proven it to be highly comparable and less time-consuming [45]. The definitions and representative images of these categories are provided in S1 File.

We grouped substratum composition into two categories: soft and hard materials. Anything larger than gravel (i.e. rubble, cobbles, boulders and bedrock) was classified as ‘hard’ material, while mud, sand and gravel were classified as ‘soft’ material according to Stein *et al*. [8]. For a given location, the sum of total hard and total soft cover (i.e. the percentage of ‘hard’ and ‘soft’ materials) was always 100%. Additionally, the presence of epibenthic communities provides additional information to correctly classify substratum. For instance, biota/benthic organisms (i.e. sessile organisms like sponges, hard corals and octocorals) that require hard substratum for growth [1, 3, 4, 47, 48] were found in amongst soft substratum according to the video data alone.

#### Seabed hardness classification

Seabed substratum is usually classified based on the video footage according to the approach by Stein *et al*. [8]. However for certain substratum compositions, it is difficult to apply. For example, for a substratum composition with 40% sand, 45% pebble and 15% gravel, it is impossible to apply this approach. Therefore, to overcome such issues we developed two new systems by modifying Stein *et al*.’s approach as below.

On the basis of Stein *et al*. [8], we developed a new system to classify the seabed substrate into four categories: hard, hard-soft, soft-hard and soft. If a substratum consisted of >70% hard material, it was classed as ‘hard’. If it consisted of ≤70% and >50% hard material, it was classed as ‘hard-soft’. If it consisted of <50% and ≥30% hard material, it was classed as ‘soft-hard’. And if it consisted of <30% hard material, it was classed as ‘soft’. This classification system is hereinafter referred to as ‘hard70’ in this study. This system would produce similar results as Stein *et al*. [8], but could avoid some issues associated with the concepts of primary and secondary substrate in Stein *et al*. [8] as discussed above.

Our initial assessment showed that our samples were mostly classified as ‘soft’, while a small number of samples were ‘hard’, leaving a limited number of samples for the mixed classes when hard70 was employed. Hence, another new classification system is proposed in this study. If a substratum consisted of ≥90% hard material, it was classed as ‘hard’. If it consisted of <90% and >50% hard material, it was classed as ‘hard-soft’. If it consisted of <50% and >10% hard material, it was classed as ‘soft-hard’. And if it consisted of ≤ 10% hard material, it was classed as ‘soft’. This system is hereinafter referred to as ‘hard90’.

In these two classification systems, a substrate consisting of 50% ‘hard’ and 50% ‘soft’ materials was assumed to be non-existent. If it indeed exists in reality, we would need to re-examine the video images and decide which one has greater cover or assign a fifth class to the system(s). However, this is not the case in this study. In total, 140 samples of seabed hardness were considered in this study. Of the 140 samples, 9 and 6 samples were recorded as hard, 11 and 14 hard-soft, 6 and 9 soft-hard and 114 and 111 soft based on hard70 and hard90 systems respectively. The resultant datasets were used to predict seabed hardness, with hardness classes based on hard90 presented in Fig 1B.

### Predictive variables

Following a preliminary analysis based on data availability and the relationships with seabed hardness as discussed above and in previous studies [7, 9–12], 41 predictive variables (i.e. features) were initially selected for this study. They are:

1 Easting,
2 Northing,
3 Bathymetry (bathy): a measure of depth of bodies of water,
4 Local Moran I of bathymetry(bathy.moran): a measure of local spatial autocorrelation in bathymetry,
5 Planar curvature (planar.curv): a curvature of the surface perpendicular to the slope direction (second derivative of bathymetry),
6 Profile curvature (profile.curv): a curvature of the surface in the direction of slope (second derivative of bathymetry),
7 Topographic relief (relief): a measure of difference between the highest and the lowest points (variance) in the surrounding cells,
8 Seabed slope (slope): slope gradient (first derivative of bathymetry),
9 Surface area (surface): the ratio of the “true” surface area and its “planar” surface area,
10 Topographic position index (tpi): a measure of difference between a cell elevation and the average of the elevation values in the surrounding cells,
11–37 Backscatter (bs10 to bs36): a diffused reflection of acoustic energy due to scattering process back to the direction from which it's been generated, measured as the ratio of the acoustic energy sent to a seabed to that returned from the seabed, normalised to incidence angles between 10° and 36°,
38 Homogeneity of backscatter (homogeneity): a measure of closeness of the distribution of elements in the Gray-Level Co-occurrence Matrix (GLCM) to the GLCM diagonal,
39 Variance of backscatter (variance): a measure of the dispersion of the values around the mean within the GLCM,
40 Local Moran I of backscatter (bs.moran): a measure of local spatial autocorrelation in backscatter, and
41 Prock: the probability of hard substrate.

The first two variables are the coordinates of sample locations. The next eight variables are bathymetry and its derived variables. The remaining 31 variables are backscatter and its derived variables. Acquisition and processing of multibeam bathymetry, backscatter and their derived variables, and prock have been detailed in previous studies [12] and in relevant online metadata [49–61]. All these variables were available at each grid cell to a 10 m resolution in the four study areas for generating the spatial predictions of seabed hardness and are freely available [49–61]. These 41 variables were also available at 140 sample locations for developing models to predict seabed hardness. The dataset for developing predictive models in this paper is provided as S2 File, where the study areas and hardness are factors and all the variables are numerical.

### Selecting predictors based on correlation analysis

There were strong correlations among some predictive variables based on Spearman’s rank correlation that was used due to non-linear relationships between some variables. We removed 21 backscatter (bs) variables that were perfectly correlated with other variables or with a *ρ* = 0.99, which is usually called a correlation-based filter FS method [28, 62]. The selection was also according to their relation with the total hard (i.e., whether they displayed a better relationship with total hard) and their correlation coefficients with other bs variables. The bs25 should have been removed according to the above selection criteria, but was retained because it was used in a previous study [7]. The Pearson’s correlation (*r*) was also derived for the remaining bs variables. The correlations of the remaining 20 variables (Table 1) and seabed hardness (i.e., hard total, the percentage cover of hard materials) were presented in Table 2.

**Table 1:** Predictive variables and their corresponding number.

No.	Predictive variable	No.	Predictive variable
1	easting	11	tpi
2	northing	12	bs13
3	prock	13	bs21
4	bathy	14	bs25
5	bathy.moran	15	bs27
6	planar.curv	16	bs32
7	profile.curv	17	bs35
8	relief	18	homogeneity
9	slope	19	variance
10	surface	20	bs.moran

**Table 2:** Spearman correlation coefficients (*ρ*) among 20 predictive variables and seabed hardness (i.e. hard total) (n = 140).

	easting	northing	prock	bathy	bathy.moran	planar.curv	profile.curv	relief	slope	surface	tpi	bs13	bs21	bs25	bs27	bs32	bs35	homogeneity	variance	bs.moran
hard.total	-0.28	0.05	0.46	-0.37	0.34	-0.07	-0.14	0.23	0.16	0.28	-0.21	0.45	0.43	0.45	0.47	0.49	0.48	0.23	-0.13	0.34
easting	1	-0.85	0.36	-0.53	0.08	-0.01	0.01	-0.3	-0.25	-0.29	0.07	0.4	0.43	0.4	0.38	0.36	0.4	0.31	-0.27	0.04
northing	-0.85	1	-0.52	0.71	-0.23	0.01	-0.01	0.17	0.2	0.15	-0.04	-0.6	-0.62	-0.61	-0.6	-0.6	-0.63	-0.43	0.35	-0.16
prock	0.36	-0.52	1	-0.81	0.48	0.06	0.03	-0.13	-0.17	-0.08	0.03	0.78	0.79	0.78	0.77	0.74	0.78	0.41	-0.3	0.45
bathy	-0.53	0.71	-0.81	1	-0.26	-0.05	0.03	0.18	0.22	0.14	0.02	-0.88	-0.89	-0.88	-0.88	-0.83	-0.87	-0.53	0.42	-0.31
bathy.moran	0.08	-0.23	0.48	-0.26	1	-0.11	0.1	0.24	0.17	0.25	0.14	0.35	0.28	0.31	0.32	0.36	0.33	0.02	0.04	0.56
planar.curv	-0.01	0.01	0.06	-0.05	-0.11	1	-0.18	-0.13	-0.13	-0.09	-0.45	0.05	0.06	0.05	0.04	0.01	0.04	0.07	-0.04	-0.09
profile.curv	0.01	-0.01	0.03	0.03	0.1	-0.18	1	0.03	-0.08	-0.01	0.65	0	-0.03	-0.03	-0.04	-0.01	-0.05	-0.13	0.13	0.08
relief	-0.3	0.17	-0.13	0.18	0.24	-0.13	0.03	1	0.88	0.92	-0.06	-0.04	-0.06	-0.02	-0.01	0	-0.04	-0.33	0.44	0.19
slope	-0.25	0.2	-0.17	0.22	0.17	-0.13	-0.08	0.88	1	0.83	-0.07	-0.07	-0.09	-0.05	-0.04	-0.03	-0.07	-0.27	0.37	0.09
surface	-0.29	0.15	-0.08	0.14	0.25	-0.09	-0.01	0.92	0.83	1	-0.08	0.02	-0.02	0.03	0.05	0.07	0.02	-0.29	0.43	0.15
tpi	0.07	-0.04	0.03	0.02	0.14	-0.45	0.65	-0.06	-0.07	-0.08	1	0	-0.03	-0.03	-0.03	-0.01	-0.05	-0.13	0.08	0.16
bs13	0.4	-0.6	0.78	-0.88	0.35	0.05	0	-0.04	-0.07	0.02	0	1	0.98	0.98	0.98	0.94	0.97	0.51	-0.43	0.21
bs21	0.43	-0.62	0.79	-0.89	0.28	0.06	-0.03	-0.06	-0.09	-0.02	-0.03	0.98	1	0.99	0.98	0.92	0.96	0.52	-0.44	0.19
bs25	0.4	-0.61	0.78	-0.88	0.31	0.05	-0.03	-0.02	-0.05	0.03	-0.03	0.98	0.99	1	0.99	0.95	0.97	0.51	-0.42	0.21
bs27	0.38	-0.6	0.77	-0.88	0.32	0.04	-0.04	-0.01	-0.04	0.05	-0.03	0.98	0.98	0.99	1	0.96	0.98	0.51	-0.42	0.22
bs32	0.36	-0.6	0.74	-0.83	0.36	0.01	-0.01	0	-0.03	0.07	-0.01	0.94	0.92	0.95	0.96	1	0.96	0.5	-0.4	0.25
bs35	0.4	-0.63	0.78	-0.87	0.33	0.04	-0.05	-0.04	-0.07	0.02	-0.05	0.97	0.96	0.97	0.98	0.96	1	0.54	-0.44	0.22
homogeneity	0.31	-0.43	0.41	-0.53	0.02	0.07	-0.13	-0.33	-0.27	-0.29	-0.13	0.51	0.52	0.51	0.51	0.5	0.54	1	-0.85	-0.06
variance	-0.27	0.35	-0.3	0.42	0.04	-0.04	0.13	0.44	0.37	0.43	0.08	-0.43	-0.44	-0.42	-0.42	-0.4	-0.44	-0.85	1	0.15
bs.moran	0.04	-0.16	0.45	-0.31	0.56	-0.09	0.08	0.19	0.09	0.15	0.16	0.21	0.19	0.21	0.22	0.25	0.22	-0.06	0.15	1

Seabed hardness (total hard) was strongly correlated with prock, bathy, and bs and its derived variables; while it was weakly correlated with northing, planar.curv, profile.curv, slope and variance (Table 2). The relationships among the 20 variables were further illustrated in S1 Fig. High prock values were typically associated with “hard” substrate (S1 Fig). In contrast, low prock values were associated with “soft” substrate. While a similar pattern was observed for backscatter, bathymetry showed an opposite pattern with a negative correlation (Table 2). These relationships were typically non-linear. These variables could potentially be good predictors of seabed hardness.

### Application of RF

Random forest, as briefly described in [7], is an ensemble machine learning method that combines many individual regression or classification trees in the following way: from the original sample, many bootstrap samples and portions of predictive variables are drawn, and an unpruned regression or classification tree is fit to each bootstrap sample using the sampled variables. From the complete forest, the status of the response variable is usually predicted either as an average of the predictions of all trees for regression or as the class with the majority vote for classification [18, 63].

The R function, *randomForest* by Liaw and Wiener [36], was employed to develop a model to predict the spatial distribution of seabed hardness. The default values of *mtry*, *ntree* and *nodesize* are often good options [21, 36] that were also observed in marine environmental sciences [7, 15], so the default values were used for these parameters.

### Feature selection

The model selection was based on a procedure developed for RF in previous studies [7, 34, 64], which involved two steps. One step was to select predictors to form a model that is often termed as feature selection, and the other was to estimate the predictive accuracy of the model formed that is addressed in the next section. To select predictive variables, we adopted the same principle used in *rfcv*, a cross-validation function in the randomForest package [36], that is, identifying and removing the least important variables based on the importance of predictive variables.

Five FS methods were used to select predictors in this study based on all 140 samples. These methods are: 1) the variable importance (VI), 2) averaged variable importance (AVI), 3) knowledge informed AVI (KIAVI), 4) Boruta and 5) RRF. The first method (i.e., VI) was based on the procedure in a previous study [7] as detailed below and was applied to hard90 data with 20 variables. For this FS method, we initially used all 20 variables to establish the full model. We then reduced the full model by gradually removing the least important variable(s) from the previous model based on the variable importance measure by RF (see Fig A in S3 File), which resulted in 22 models (see Table 3). Two exceptions to this are that: 1) for model 18 and 19, since bs25 and bs27 are equally important, we excluded them from model 17 respectively; and 2) for model 22, we included it because prock was the most important predictor in a previous study [7]. After reaching the model with minimum number of predictors (i.e. only one predictor remained), we then identified the important predictor(s) based on the PA of the models developed. The important predictor(s) were defined as follows: if their exclusion reduced the PA of the subsequent model, they were determined to be important (i.e. variance, surface and relief). We also identified the unimportant predictor(s) (i.e. bs27) that increased the PA when they were excluded. We then repeated above procedure by adding these important predictors and removing the unimportant predictor to the most accurate model so far (i.e., model14) to develop predictive models until no further improvement in the PA could be achieved.

**Table 3:** A brief summary of RF modelling process for hard90 data using various FS methods and predictive variables. 1) models 1–25 based on the VI using 20 variables; 2) models 26–29 based on the AVI using 20 variables; 3) models 30–31 based on KIAVI using 20 variables; 4) models 32–43 based on the AVI using 41 variables; and 5) models 44–45 based on the Boruta and model 46 based on the RRF using 41 variables. Model.fit is the predictive accuracy (*ccr*) of training samples by each RF model developed. The corresponding predictor for each number is listed in Table 1.

Model	Modelling.process	Predictors	No.predictors	Model.fit
1	All 20 predictive variables	All 20 variables	20	100
2	model 1:—planar.curv	1–5,7–20	19	100
3	model 2:—surface	1–5, 7–9, 11–20	18	100
4	model 3:—slope	1–5, 7–8, 11–20	17	100
5	model 4:—relief	1–5, 7, 11–20	16	100
6	model 5:—bathy.moran	1–4, 7, 11–20	15	100
7	model 6:—profile.curv	1–4, 11–20	14	100
8	model 7:—bs.moran	1–4, 11–19	13	100
9	model 8:—bathy	1–3, 11–19	12	100
10	model 9:—homogeneity	1–3, 11–17, 19	11	100
11	model 10:—variance	1–3, 11–17	10	100
12	model 11:—northing	1, 3, 11–17	9	100
13	model 12:—tpi	1, 3, 12–17	8	100
14	model 13:—bs13	1, 3, 13–17	7	100
15	model 14:—bs21	1, 3, 14–17	6	100
16	model 15:—easting	3, 14–17	5	100
17	model 16:—bs32	3, 14–15, 17	4	100
18	model 17:—bs27	3, 14, 17	3	96.43
19	model 17:—bs25	3, 15, 17	3	96.43
20	model 18:—bs25	3, 17	2	96.43
21	model 20:—prock	17	1	100
22	model 20:—bs35	3	1	91.43
23	model 14: + variance	1, 3, 13–17, 19	8	100
24	model 23: + surface	1, 3, 10, 13–17, 19	9	100
25	model 24: + relief	1, 3, 8, 10, 13–17, 19	10	100
26	Six most important predictors	1, 10, 16–19	6	100
27	model 26: + prock and bs27	1, 3, 10, 15–19	8	100
28	model 27: + planar.curv	1, 3, 6, 10, 15–19	9	100
29	model 27:—prock	1, 10, 15–19	7	100
30	Combine model 24 and 27	1, 3, 10, 13–19	10	100
31	model 30:—bs21	1, 3, 10, 14–19	9	100
32	The 13 predictors most important	1, 10, 18, 19, bs28-bs36	13	100
33	model 32: + bs10	1, 10, 18, 19, bs10, bs28-bs36	14	100
34	model 33: + planar.curv	1, 6, 10, 18, 19, bs10, bs28-bs36	15	100
35	model 34: + northing	1, 2, 6, 10, 18, 19, bs10, bs28-bs36	16	100
36	model 35: + prock, bs.moran, bs27	1–3, 6, 10, 18–20, bs10, 15, bs28-bs36	19	100
37	model 36:—bs27	1–3, 6, 10, 18–20, bs10, bs28-bs36	18	100
38	model 37:—bs.moran	1–3, 6, 10, 18, 19, bs10, bs28-bs36	17	100
39	model 37:—prock	1, 2, 6, 10, 19–20, bs10, bs28-bs36	17	100
40	model 35:—planar.curv	1, 2, 10, 18, 19, bs10, bs28-bs36	15	100
41	model 40:—northing	1, 10, 18–19, bs10, bs28-bs36	14	100
42	model 41:—bs34	1, 2, 10, 17–19, bs10, bs28-bs33, bs36	14	100
43	All 41 predictors	All 41 predictors	41	100
44	30 predictors	1–3, 7, 11, 20, bs12, bs14:bs36	30	100
45	model 44: +bs13	1–3, 7, 11, 20, bs12:bs36	31	100
46	31 predictors	1–3, 5, 7–11, 18–20, bs15, bs17-bs18, bs20-bs25, bs27-bs36	31	100

We then applied the second model selection method (i.e., AVI). Due to the randomness associated with the importance of predictive variables generated by RF algorithm, the least important variable(s) may change with individual iterations; meanwhile, correlated variables may also affect the order of the least important variable(s); so an R package ‘*extendedForest*’ [39] was used and repeated 100 times to generate the average values of variable importance (Fig B in S3 File) that were used to select the predictors. This approach was applied to hard90 using 20 variables, which led to four models (i.e. models 26 to 29; see Table 3). We initially selected the most importance predictors when the level was 4 or 6 according to Fig B in S3 File, then added the next important predictor(s) until no further improvement in PA was gained. We then removed the least important predictor from the most accurate model to determine if further improvement was possible.

We also used KIAVI (i.e. applied AVI to a combined model that was based on two most accurate models identified via VI and AVI using 20 predictive variables) to see if we could further improve the accuracy. Since AVI can deal with correlated variables, we also applied this approach to the whole dataset (i.e. using 41 variables) (Fig C in S3 File) by adopting the rationale for adding and removing predictor(s) as stated above.

We also applied AVI to hard70 data using 20 and 41 variables. We also applied AVI to a combined model based on the most accurate model identified for hard90 and the most accurate model based on AVI approach using 20 variables for hard70 to see if we could further improve the PA, which is also a kind of KIAVI and hereinafter referred to as the ‘KIAVI’.

We then used Boruta [40] to search for the important predictors for hard90 and hard70 data using all 41 variables because it is an all-relevant FS algorithm. For hard90 data, the default value (i.e., 100) was used for the maximal number of importance source runs in the final round. To resolve predictive variables left as ‘Tentative’, we increased the number of importance source runs to 2000 and 5000 respectively, but they selected the same variables. For hard70 data, we used the default value as well as the values of 2000 and 5000 for the maximal number of importance source runs. The selected variables were then used in the *randomForest* function.

Lastly, we used RRF [41] to search the important predictors for hard90 and hard70 data using 41 variables because it is also an all-relevant FS algorithm. This is hereinafter referred to as RRF approach in this study. The selected variables were then used in the *randomForest* function.

### Model validation

To identify the most accurate predictive model, we need to know the PA of each model formed from the above FS methods. To achieve this, we used *rf*.*cv* that validates one model with fixed predictive variables for all iterations for a given number of predictive variables [7]. This function allows variations in datasets generated by cross-validation and ensures the model select relevant predictors from a list of the fixed predictive variables. Given that the response variable is categorical, the correct classification rate (*ccr*) [65] and *kappa* [66] were used to measure the accuracy of the predictive model and were calculated using the built-in functions in *rf*.*cv*.

To assess the predictive ability of each model, we used 10-fold cross-validation [67]. To deal with the random error associated with each 10-fold cross validation [7, 34, 64], the cross validation procedure was repeated 100 times. The choice of this iteration number was based on findings in previous studies [7, 34] and that the dynamics of the predictive accuracies with iterations of relevant models in this study suggested that averaged accuracies stabilised after 20–80 iterations. The final results were based on the average of 100 iterations of the cross validation.

### Model comparison and spatial predictions

Since the data of *ccr* and *kappa* were not normally distributed based on the Shapiro-Wilk normality test, with heterogeneous variance based on Fligner-Killeen test of homogeneity of variances, or both, Mann-Whitney tests were used to compare the PA in terms of *ccr* and *kappa* between the most accurate models for both hard90 and hard70 data.

Finally, the most accurate predictive models for hard90 and hard70 data were used to predict seabed hardness at each 10m grid cell in the study areas. A portion of area A (A1) that comprises a variety of seabed geomorphic features was selected to illustrate and compare the predictions.

All relevant computing work was implemented in R 2.15.2 [68]. Relevant maps were then produced using ArcGIS (ESRI ® ArcMap ^TM^ 10.0) [69].

## Results

### Predictive model for hard90 data

#### Model selection using VI for 20 predictive variables (VI & filter)

**Fig 2:**
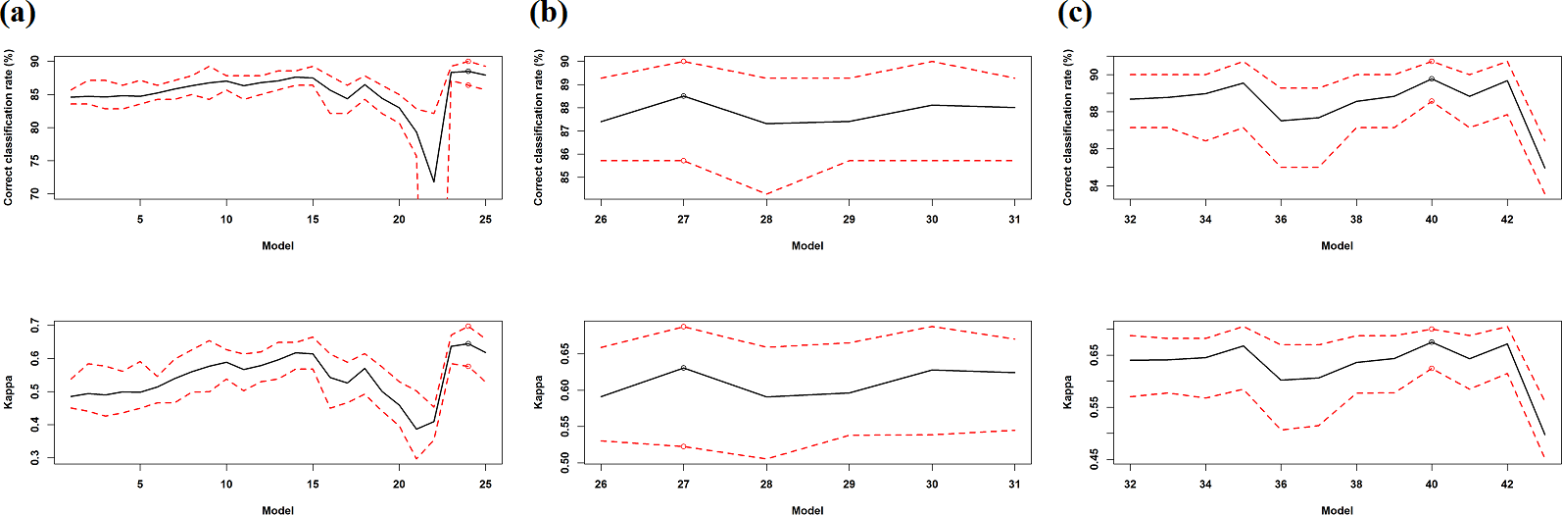
Correct classification rate (%) and kappa (mean: black line; minimum and maximum: dash red lines) of 43 RF models with different predictor sets based on the averages over 100 iterations of 10-fold cross validation for seabed hardness based on hard90 data; and the model with the maximum mean ccr and mean kappa (circle). a) models 1–25 based on the VI using 20 predictive variables; b) models 26–29 based on the AVI and models 30–31 based on KIAVI using 20 variables; c) models 32–43 based on the AVI using 41 variables.

In total, 25 models were developed based on the model selection approach using variable importance of 20 variables (models 1–25 in Table 3, Fig 2A). Correct classification rates gradually increased from model 1 and reached a maximum mean (i.e. 87.64%) for model 14, except that the PA of models 3, 5 and 11 slightly decreased after the removal of surface, relief and variance respectively. It then began to decrease from model 15 onwards with an abrupt increase for model 18 due to the exclusion of bs27, and reached the lowest value for model 22 that contained only one predictor. After adding variance and surface to model 14, the PA was further improved and reached the highest mean value of 88.53% for model 24 (Fig 2A, Table 3). *Kappa* displayed a similar pattern as *ccr* and reached the highest mean value of 0.6449 for model 24, with the exception of that the lowest value was attained for model 21 that again contained only one predictor. It showed that bs35 was the most important predictor based on *ccr* while prock was the most important predictor based on *kappa*. Overall, model 24 was more accurate than other models in terms of both of *ccr* and *kappa*. This model contained nine predictors (Fig 2A, Table 3). In addition, most models perfectly predicted the training samples. Removing bs27 from model 24 did not improve the accuracy, so it was not presented in the results.

#### Model selection using AVI and KIAVI for 20 predictive variables (AVI & filter and KIAVI & filter)

On the basis of the AVI using 20 variables, further four models were developed (models 26–29 in Table 3, Fig 2B). Correct classification rates reached the highest mean value of 88.51% for model 27 after the inclusion of bs27 and prock. It then started to decrease after adding planar.curv (i.e. model 28) and excluding prock (i.e. model 29). It showed that adding or excluding a further variable reduced the PA of model 27. *Kappa* displayed an identical pattern to *ccr* and reached the highest mean value of 0.6304 for model 27. Combing the two best performing models identified so far (i.e. models 24 and 27) resulted in two further models (models 30 and 31 in Table 3) that did not improve the PA in comparison with model 27. Overall, model 27, containing eight predictors, was more accurate than other models in terms both of *ccr* and *kappa* (Fig 2B, Table 3).

#### Model selection using AVI for 41 predictive variables

On the basis of the AVI using 41 variables, a further 12 model were developed (models 32–43 in Table 3, Fig 2C). The PA increased after adding bs10, planar.curv and northing, which resulted in a highly accurate model (i.e. model 35). After adding the next three most important predictors, the PA decreased. Further tuning to model 36 by removing the next less important predictor(s) resulted in models 37 to 39. The PA of all these models was less than that of model 35. Further tuning to model 35 by removing the least important predictor (i.e. planar curv) resulted in the most accurate model 40 (with a mane *ccr* of 89.78%). Model 43, which used all 41 variables, was much less accurate than all other models (Fig 2C). *Kappa* displayed an identical pattern as *ccr* and reached the highest mean value of 0.6753 for model 40. Overall, model 40 was relatively more accurate than other models in terms both of *ccr* and *kappa* and contained 15 predictors (Table 3). In addition, model 40 could not be further improved by removing either the next less important predictor (model 41) or the highly correlated predictor (model 42).

#### Model selection using Boruta for 41 predictive variables

On the basis of the Boruta approach, two models were developed (models 44–45 in Table 3). Model 44 contained 30 predictors with a mean *ccr* of 85.79% (ranging from 85% to 86.43%) and a mean *kappa* of 0.5279 (ranging from 0.4897 to 0.5616). Model 45 contained 31 predictors with a mean *ccr* of 85.83% (ranging from 85% to 87.14%) and a mean *kappa* of 0.5301 (ranging from 0.4986 to 0.5845), which was slightly more accurate than model 44.

#### Model selection using RRF for 41 predictive variables

On the basis of the RRF approach, a further model was developed (model 46 in Table 3). Model 46 contained 31 predictors with a mean *ccr* of 85.27% (ranging from 83.57% to 87.14%) and a mean *kappa* of 0.5078 (ranging from 0.4511 to 0.5853).

In summary, 46 models were developed for hard90 data. Model 24 was the most accurate model based on the VI and model 27 was the most accurate model based on the AVI using 20 variables; and model 40 was the most accurate model based on the AVI and model 45 was the most accurate model based on the Boruta using 41 variables. Overall, model 40 was the most accurate model.

#### Agreement of the observed and predicted values of the most accurate model

The predicted values based on the most accurate model (i.e., model 40) and the observed values matched well for most classes in terms of both user’s accuracy and producer’s accuracy, although producer’s accuracy was poor for the hard-soft class (Table 4). When the hard-soft and soft-hard classes were merged into the hard class, the accuracies were improved, especially for the user’s accuracy for the hard class (Table 5). The user’s accuracy was higher than the producer’s accuracy for non-soft classes (Tables 4 and 5). Non-soft classes, particularly hard-soft, were under-predicted while the soft class was over-predicted.

**Table 4:** Confusion matrix between the observed and predicted values of four hardness classes based on the average of 100 times of 10-fold cross validation using the most accurate predictive model (i.e., model 40) for hard90.

		Observed					
		Hard	Hardsoft	Softhard	Soft	Total	User's accuracy
Predicted	Hard	4	1	0.42	0	5.42	73.80
	Hardsoft	0	6.84	0.86	1.89	9.59	71.32
	Softhard	0	0	5.87	0.13	6	97.83
	Soft	2	6.16	1.85	108.98	118.99	91.59
	Total	6	14	9	111	140	
	Producer's Accuracy	66.67	48.86	65.22	98.18		89.78

**Table 5:** Confusion matrix between the observed and predicted values of two hardness classes based on the average of 100 times of 10-fold cross validation using the most accurate predictive model (i.e., model 40) for hard90.

		Observed			
		Hard	Soft	Total	User's accuracy
Predicted	Hard	18.99	2.02	21.01	90.39
	Soft	10.01	108.98	118.99	91.59
	Total	29	111	140	
	Producer's Accuracy	65.48	98.18		91.41

### Predictive model for hard70 data

#### Model selection using AVI for 20 predictive variables (AVI & filter)

**Fig 3:**
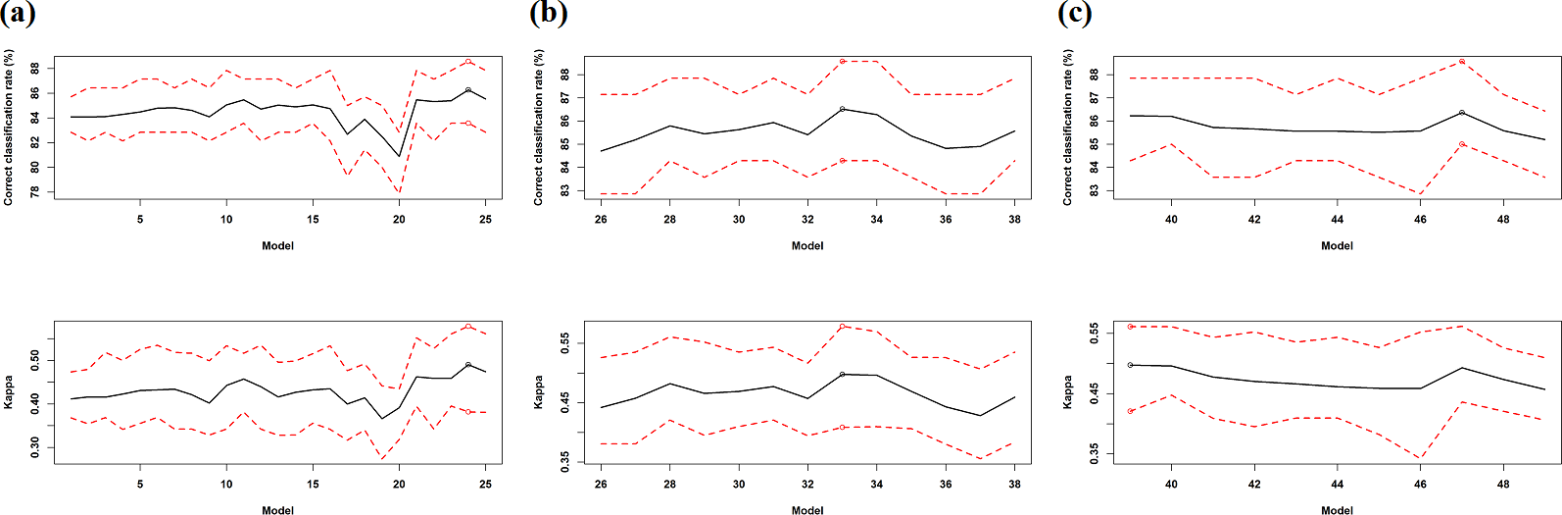
Correct classification rate (%) and kappa (mean: black line; minimum and maximum: dash red lines) of 49 RF models with different predictor sets based on the averages over 100 iterations of 10-fold cross validation for seabed hardness based on hard70 data; and the model with the maximum mean ccr and mean kappa (circle). a) models 1–25 based on the AVI using 20 predictive variables; b) models 26–38 based on the AVI using 41 variables; c) models 39–49 based on KIAVI using 41 variables.

Twenty five models were developed based on the AVI for 20 variables (models 1–25 in Table 6, Fig 3A). The first twenty models were developed by removing the least important variable based on AVI. Correct classification rates reached a local maximum for model 11. Two predictors (i.e. bs21 and bathy.moran) were identified as important variables and a few predictors were identified as unimportant variables (e.g. profile.curv, bs.moran, variance).

**Table 6:** A brief summary of RF modelling process for hard70 data using various FS methods and predictive variables. 1) models 1–25 based on the AVI using 20 variables; 2) models 26–38 based on the AVI using 41 variables; 3) models 39–49 based on KIAVI using 41 variables; and 4) models 50–52 based on the Boruta with the maximal number of importance source runs of 2000, 100 and 5000, and model 53 based on the RRF using 41 variables. The model fit is the predictive accuracy (*ccr*) of training samples by each RF model developed. The corresponding predictor for each number is listed in Table 1.

Model	Modelling.process	Predictors	No.predictors	Model.fit
1	All 20 predictive variables	All 20 variables	20	100
2	model 1: -relief	1–7, 9–20	19	100
3	model 2: -northing	1, 3–7, 9–20	18	100
4	model 3: -bs13	1, 3–7, 9–11, 13–20	17	100
5	model 4: -bs27	1, 3–7, 9–11, 13–14, 16–20	16	100
6	model 5: -slope	1, 3, 4–7, 10–11, 13–14, 16–20	15	100
7	model 6: -bs25	1, 3–7, 10–11, 13, 16–20	14	100
8	model 7: -bs21	1, 3–7, 10–11, 16–20	13	100
9	model 8: -bathy.moran	1, 3–4, 6–7, 10–11, 16–20	12	100
10	model 9: -surface	1, 3–4, 6–7, 11, 16–20	11	100
11	model 10: -bathy	1, 3, 6–7, 11, 16–20	10	100
12	model 11: -tpi	1, 3, 6–7, 16–20	9	100
13	model 12: -bs35	1, 3, 6–7, 16, 18–20	8	100
14	model 13: -variance	1, 3, 6–7, 16, 18, 20	7	100
15	model 14: -bs.moran	1, 3, 6–7, 16, 18	6	100
16	model 15: -bs32	1, 3, 6–7, 18	5	100
17	model 16: -easting	3, 6–7, 18	4	100
18	model 17: -profile.curv	3, 6, 18	3	96.43
19	model 18: -homogeneity	3, 6	2	95
20	model 19: -planar.curv	3	1	91.43
21	model 11: +bs21	1, 3, 6–7, 11, 13, 16–20	11	100
22	model 21: +bathy.moran	1, 3, 5–7, 11, 13, 16–20	12	100
23	model 21: -profile.curv	1, 3, 6, 11, 13, 16–20	10	100
24	model 21: -bs.moran	1, 3, 6–7, 11, 13, 16–19	10	100
25	model 24: -variance	1, 3, 6, 7, 11, 18, 13, 16, 17	9	100
26	All 41 predictors	1–11, 18–20, bs10-bs36	41	100
27	model 26:—bs25, bs10:bs14	1–11, 18–20, bs15-bs36	35	100
28	model 27:—bathy, relief, bs24,bs26, bs27	1–3, 5–7, 9–11, 18–20, bs15-bs23, bs28-bs36	30	100
29	model 28:—bs20, bs29, bs30, bs36	1–3, 5–7, 9–11, 18–20, bs15-bs19, bs21-bs23, bs28, bs31-bs35	26	100
30	model 29:—nothing and bs28	1, 3, 5–7, 9–11, 18–20, bs15-bs19, bs21-bs23, bs31-bs35	24	100
31	model 30:—slope and bs34	1, 3, 5–7, 10, 11, 18–20, bs15 to bs19, bs21-bs23, bs31-bs33, 17	22	100
32	model 31-—bs15, bs16, bs19, bs21, bs32, bs33 and bs 35	1, 3, 5–7, 10, 11, 18–20, bs17, bs18, bs22, bs23, bs31	15	100
33	model 32-—bathy.moran, surface, bs17 and bs22	1, 3, 6–7, 11, 18–20, bs18, bs23, bs31	11	100
34	model 33-—homogeneity	1, 3, 6–7, 11, 19–20, bs18, bs23, bs31	10	100
35	model 34-—tpi	1, 3, 6–7, 19–20, bs18, bs23, bs31	9	100
36	model 35-—bs23	1, 3, 6–7, 19–20, bs18, bs31	8	100
37	model 36-—bs18	1, 3, 6–7, 19–20, bs31	7	100
38	model 37-—bs.moran	1, 3, 6–7, 19, bs31	6	100
39	Variables for model 24, 33, and model 40 for hard90	1–3, 6–7, 10–11, 13, 18–20, bs10, bs18, bs23, bs28-bs36	23	100
40	model 39-—bs10	1–3, 6–7, 10–11, 13, 18–20,bs18, bs23, bs28-bs36	22	100
41	model 40-—bs28, bs30 abd bs36	1–3, 6–7, 10–11, 13, 18–20, bs18, bs23, bs29, bs31-17	19	100
42	model 41-—northing	1, 3, 6–7, 10–11, 13, 18–20, bs18, bs23, bs29, bs31-17	18	100
43	model 42-—bs35	1, 3, 6–7, 10–11, 13, 18–20, bs18, bs23, bs29, bs31-bs34	17	100
44	model 43-—bs29 and bs34	1, 3, 6–7, 10–11, 13, 18–20,bs18, bs23, bs31-bs33	15	100
45	model 44-—bs21	1, 3, 6–7, 10–11, 18–20,bs18, bs23, bs31-bs33	14	100
46	model 45-—bs33	1, 3, 6–7, 10–11, 16, 18–20,bs18, bs23, bs31	13	100
47	model 46-—surface	1, 3, 6–7, 11, 16, 18–20,bs18, bs23, bs31	12	100
48	model 47-—tpi	1, 3, 6–7, 16, 18–20,bs18, bs23, bs31	11	100
49	model 47-—tpi and bs31	1, 3, 6–7, 16, 18–20, bs18, bs23	10	100
50	27 predictors	1, 3, 7, 11, bs12, bs13, bs16-bs36	27	100
51	model 50-—easting, bs12	2–3, 7, 11, 20, bs13, bs16-bs36	25	100
52	model 50- +bs10, bs14	1, 3, 7, 11, bs10, bs12-bs14, bs16-bs36	29	100
53	31 predictors	1–3, 5, 7–11, 18–20, bs15, bs17-bs18, bs20-bs25, bs27-bs36	31	100

After further adding the important variables to model 11 and removing the unimportant predictors from subsequent models, *ccr* increased and reached the highest mean value of 86.27% for model 24. *Kappa* displayed a similar pattern as *ccr* and reached the highest mean value of 0.4905 for model 24. Overall, model 24 with 10 predictors was more accurate than other models. In addition, most models perfectly predicted the training samples.

#### Model selection using AVI and KIAVI for 41 predictive variables

On the basis of the AVI for 41 variables, a further 13 models were developed (models 26–38 in Table 6, Fig 3B). The AVI of model 38 for hard70 data using 41 variables showed that easting should be excluded, while on the basis of model 24 for hard70 data it should be included. Hence no further model development was conducted for hard70 data using 41 variables. The most accurate model was model 33 with a mean *ccr* of 86.52%. *Kappa* displayed an identical pattern as *ccr* and reached the highest mean value (i.e. 0.4976) for model 33. This model contained 11 predictors.

On the basis of model 24 for hard70 and also model 40 for hard90 (i.e. the most accurate model), we further tuned model 33 by including additional predictors that were used in these two models. This resulted in a further 11 models (models 39–49 in Table 6). Model 39 was the most accurate model in terms of *kappa* (i.e. 0.4973) while model 47 is the most accurate model in terms of *ccr* (i.e. 86.36) (Table 6 and Fig 3C). Overall, model 33 was relatively more accurate than other models.

#### Model selection using Boruta for 41 predictive variables

On the basis of the Boruta approach, three models were developed (models 50–52 Table 6). Model 50 contained 25 predictors with a mean *ccr* of 87.04% (ranging from 85.71% to 87.86%) and a mean *kappa* of 0.5328 (ranging from 0.4836 to 0.5609). Model 51 contained 27 predictors with a mean *ccr* of 86.85% (ranging from 84.29% to 87.86%) and a mean *kappa* of 0.5318 (ranging from 0.4538 to 0.5692). Model 52 contained 29 predictors with a mean *ccr* of 86.99% (ranging from 84.29% to 87.86%) and a mean *kappa* of 0.5309 (ranging from 0.4434 to 0.5609). Model 50 was slightly more accurate than model 51 and model 52.

#### Model selection using RRF for 41 predictive variables

On the basis of the RRF, one model was developed (model 53 in Table 6). Model 53 contained 31 predictors with a mean *ccr* of 85.14% (ranging from 82.86% to 87.14%) and a mean *kappa* of 0.4533 (ranging from 0.3949 to 0.5351).

In summary, 53 models were developed for hard70 data. Model 24 was the most accurate model based on AVI using 20 variables, and model 33 was the most accurate model based on AVI and model 50 was the most accurate model based on Boruta using 41 variables. Of these 53 models, model 50 was the most accurate model.

#### Agreement of the observed and predicted values of the most accurate model

The predicted values based on the most accurate model (i.e., model 50) and the observed values matched very well for the soft class in terms of both user’s accuracy and producer’s accuracy; and producer’s accuracy was poor for non-soft classes, especially for the hard-soft class (Table 7). When the hard-soft and soft-hard classes were merged into the hard class, the accuracies were improved, especially the user’s accuracy for the hard class (Table 8). The user’s accuracy was higher than the producer’s accuracy for non-soft classes (Tables 7 and 8). All non-soft classes were under-predicted while the soft class was over-predicted.

**Table 7:** Confusion matrix between the observed and predicted values of four hardness classes based on the average of 100 times of 10-fold cross validation using the most accurate predictive model (i.e., model 50) for hard70.

		Observed					
		Hard	Hardsoft	Softhard	Soft	Total	User's accuracy
Predicted	Hard	4	1.22	0.01	2.08	7.31	54.72
	Hardsoft	1.02	4.22	0	1.24	6.48	65.12
	Softhard	0	0.3	3	0.04	3.34	89.82
	Soft	3.98	5.26	2.99	110.64	122.87	90.05
	Total	9	11	6	114	140	
	Producer's Accuracy	44.44	38.36	50.00	97.05		87.04

**Table 8:** Confusion matrix between the observed and predicted values of two hardness classes based on the average of 100 times of 10-fold cross validation using the most accurate predictive model (i.e., model 50) for hard70.

		Observed			
		Hard	Soft	Total	User's accuracy
Predicted	Hard	13.77	3.36	17.13	80.39
	Soft	12.23	110.64	122.87	90.05
	Total	26	114	140	
	Producer's Accuracy	52.96	97.05		88.86

### Comparison of FS methods based on the most accurate predictive models identified for hard90 and hard70 data

**Fig 4:**
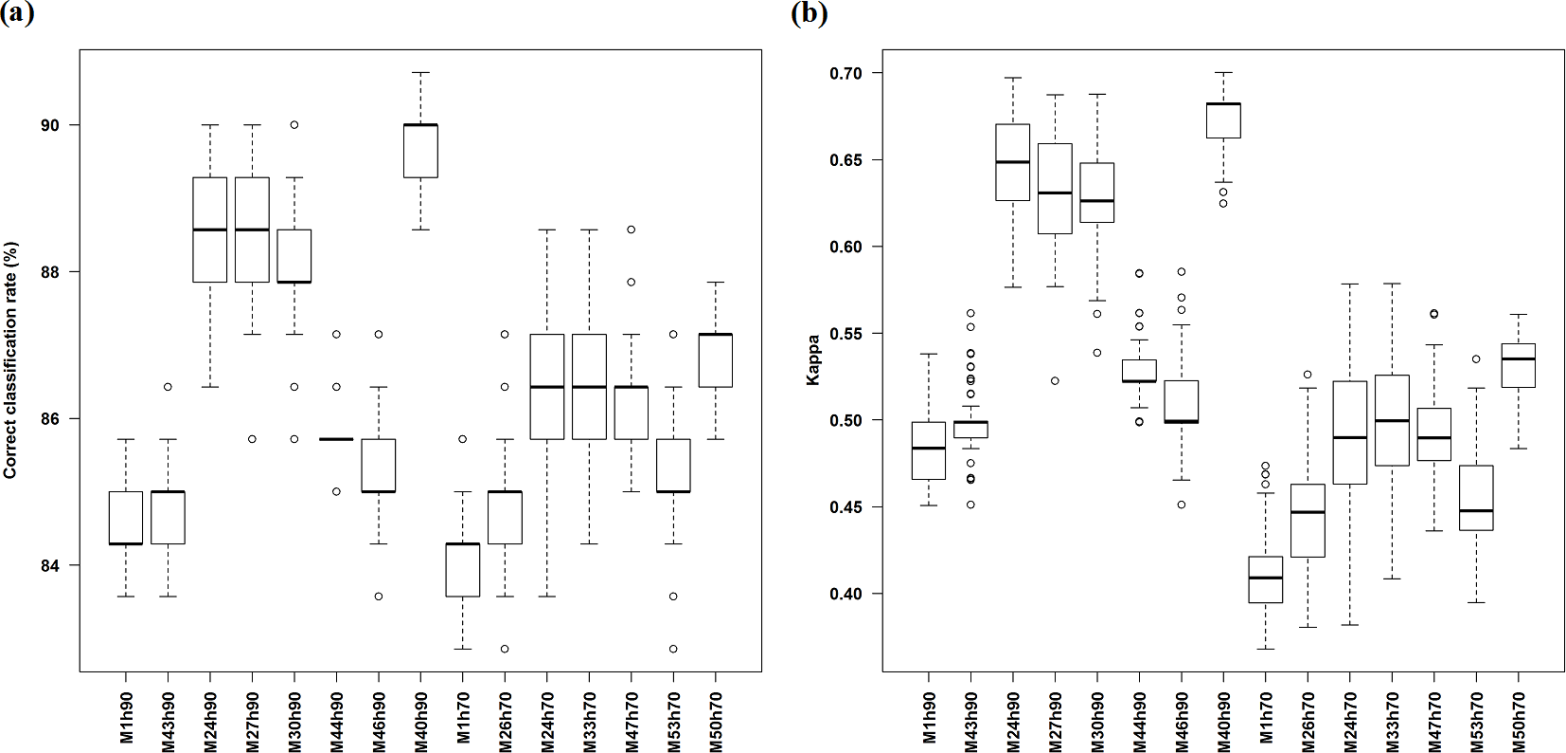
Correct classification rate (%) (a) and *kappa* (b) of the most accurate models based on the averages over 100 iterations of 10-fold cross validation for hard90 and hard70 data.

The accuracy of full models (i.e. model 43 for hard90, and model 26 for hard70) and the most accurate models identified based on various FS methods have been summarised in Table 9 and Fig 4. The models developed from 41 variables were more accurate than the models from the pre-selected 20 variables for both hard90 and hard70 (Table 9). The most accurate models based on various FS techniques were significantly more accurate than the models using either all 41 variables or the pre-selected 20 variables in terms of both *ccr* and *kappa* for both hard90 and hard70 (Table 9).

**Table 9:** Comparison of the accuracy of full models (i.e. model 43 for hard90, and models 26 for hard70) with the most accurate models based various FS methods. The differences between these comparisons based on the Mann-Whitney tests (n = 100 for each model).

Data	Model	FS method	ccr			kappa		
			ccr	*models*	*p*-value	kappa	*models*	*p*-value
Hard90	43	41 variables	84.97			0.4973		
	1	Filter (20 variables)	84.62	1 vs. 43	0.0000	0.4852	1 vs. 43	0.0000
	24	VI & filter	88.53	24 vs. 1	0.0000	0.6449	24 vs. 1	0.0000
	27	AVI & filter	88.51	27 vs. 1	0.0000	0.6305	27 vs. 1	0.0000
	30	KIAVI	88.11	30 vs. 1	0.0000	0.6278	30 vs. 1	0.0000
	40	AVI	89.78	40 vs. 43	0.0000	0.6753	40 vs. 43	0.0000
	45	Boruta	85.83	44 vs. 43	0.0000	0.5301	44 vs. 43	0.0000
	46	GRF	85.28	46 vs. 43	0.0010	0.5078	46 vs. 43	0.0013
Hard70	26	41 variables	84.71			0.4421		
	1	Filter	84.09	1 vs. 26	0.0000	0.4116	1 vs. 26	0.0000
	24	AVI & Filter	86.27	24 vs. 1	0.0000	0.4905	24 vs. 1	0.0000
	33	AVI	86.52	33 vs. 26	0.0000	0.4976	33 vs. 26	0.0000
	47	KIAVI	86.36	47 vs. 26	0.0000	0.4927	47 vs. 26	0.0000
	50	Boruta	87.04	50 vs. 26	0.0000	0.5328	50 vs. 26	0.0000
	53	GRF	85.14	53 vs. 26	0.0002	0.4533	53 vs. 26	0.0028

The most accurate models based on the FS methods were compared in Table 10 and Fig 4. For hard90, models 24 and 27 were not significantly different in terms of *ccr* and models 27 and 30 were not significantly different in terms of *kappa*. For hard70, models 24, 33 and 47 were not significantly different. All other models were significantly different in terms of PA. Their accuracy changed with FS methods in the following order: for hard90, AVI > VI & filter > AVI & filter > KIAVI & filter > Boruta > RRF; and for hard70, Boruta > AVI, AVI & filter, KIAVI > RRF.

**Table 10:** Comparison of the accuracy of the most accurate models (i.e. model 40 for hard90 and model 50 for hard70) with the most accurate models based various FS techniques, and also model 40 with model 50. The differences between these comparisons based on the Mann-Whitney tests (n = 100 for each model).

FS method Hard90	Models		*p*-value for ccr					*p*-value for kappa				
	Developed	Model	24	27	30	45	46	24	27	30	45	46
VI & filter	25	24										
AVI & filter	4	27	0.8881					0.0010				
KIAVI	2+25+4	30	0.0003	0.0006				0.0000	0.4450			
Boruta	2	45	0.0000	0.0000	0.0000			0.0000	0.0000	0.0000		
GRF	1	46	0.0000	0.0000	0.0000	0.0000		0.0000	0.0000	0.0000	0.0000	
AVI	11	40	0.0000	0.0000	0.0000	0.0000	0.0000	0.0000	0.0000	0.0000	0.0000	0.0000
Hard70		Model	24	33	47	53		24	33	47	53	
AVI & filter	25	24										
AVI	13	33	0.1079					0.3001				
KIAVI	11+25+13	47	0.6253	0.1820				0.9357	0.2328			
GRF	1	53	0.0000	0.0000	0.0000			0.0000	0.0000	0.0000		
Boruta	3	50	0.0000	0.0000	0.0000	0.0000		0.0000	0.0000	0.0000	0.0000	
Hard90/Hard70		Model	40					40				
		50	0.0000					0.0000				

The *ccr* and *kappa* of the most accurate model (i.e. model 40) for hard90 were significantly higher than the rest five models (i.e. models 24, 27, 30, 44 and 46) based on the Mann-Whitney tests (with *p* values < 0.0001). The *ccr* and *kappa* of the most accurate model (i.e. model 50) for hard70 were significantly higher than those of the remaining four models (i.e. models 24, 33, 47 and 53) based on the Mann-Whitney tests (with *p* values < 0.0001). In addition, the most accurate model for hard90 was significantly more accurate than the most accurate model for hard70 based on the Mann-Whitney test (with a *p* value < 0.0001).

In terms of computing efficiency as measured by the number of models developed for identifying the most accurate model for each FS method, RRF and Boruta were much higher than AVI, VI and KIAVI.

### Comparison of spatial predictions of seabed hardness based on hard90 and hard70 data

The spatial predictions of the most accurate models for hard90 and hard70 were similar, with a match rate between corresponding hardness classes as high as 92.31% (i.e. with a corresponding mismatch rate of 7.69%, Table 11). Hard and soft were predicted less often, while hard-soft and soft-hard were predicted in greater number based on hard90 data than hard70. Of the mismatched predictions, about 1.3% of hard predictions for hard70 were predicted as hard-soft and soft-hard for hard90, while about 5.18% of soft predictions for hard70 were predicted as hard-soft and soft-hard for hard90, leading low match rates for certain classes between hard90 and hard70.

**Table 11:** Confusion matrix between predictions for individual classes based on hard90 and hard70 data for all study areas and for a portion of area A (A1).

		All four areas- Hard70 (17,418,262 grid cells)					Correctly matched by hard70 (%)
		Hard	Hard-soft	Soft-hard	Soft	Total	
Hard90	Hard	0.85	0.23	0.00	0.09	1.17	72.60
	Hard-soft	1.26	3.68	0.10	2.06	7.12	51.77
	Soft-hard	0.04	0.13	2.65	3.12	5.95	44.60
	Soft	0.41	0.06	0.17	85.12	85.77	99.25
	Total	2.57	4.10	2.93	90.41	100.00	
Correctly matched by hard90 (%)		33.15	89.91	90.56	94.16		92.31
		Area A1- Hard70 (3,083,153 grid cells)					Correctly matched by hard70 (%)
		Hard	Hard-soft	Soft-hard	Soft	Total	
Hard90	Hard	2.01	0.12	0	0.46	2.59	77.78
	Hard-soft	5.24	14.82	0.29	6.66	27.01	54.85
	Soft-hard	0	0.14	2.45	7.17	9.76	25.09
	Soft	1.62	0.01	0.21	58.8	60.64	96.96
	Total	8.87	15.09	2.95	73.09	100	
Correctly matched by hard90 (%)		22.66	98.22	82.90	80.45		78.07

**Fig 5:**
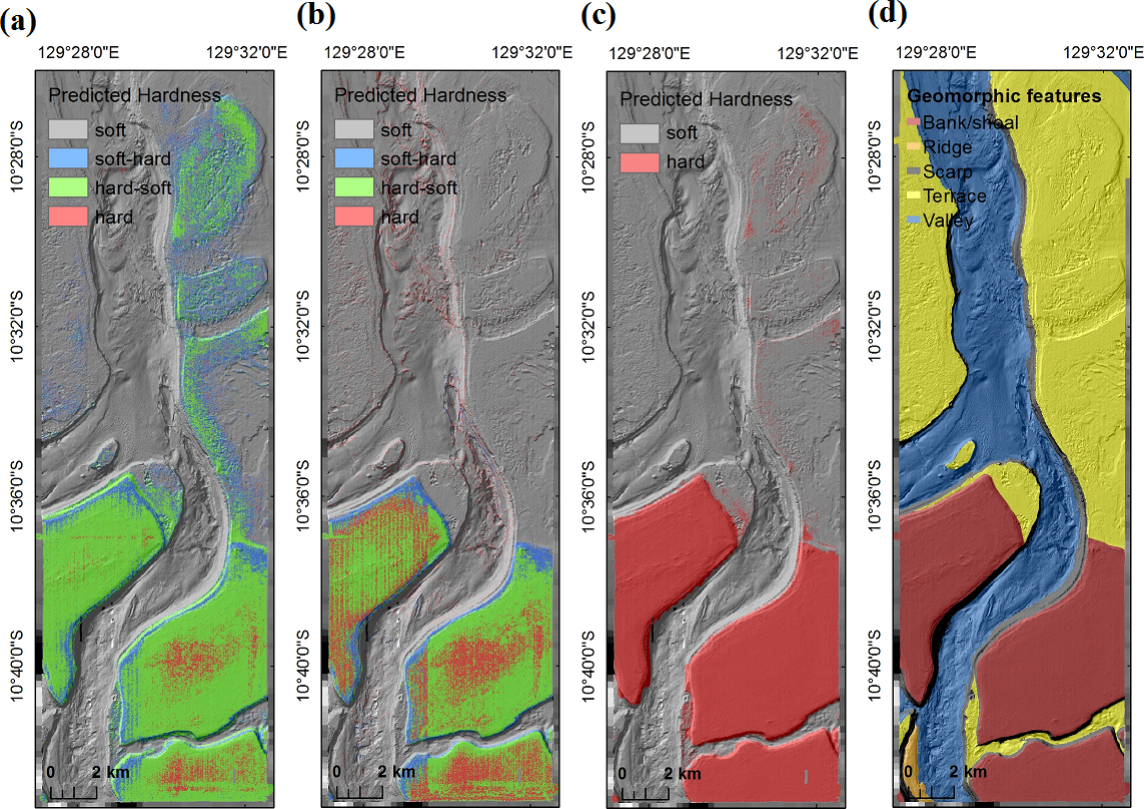
Spatial predictions of seabed hardness for a section of area A (A1): a) hard90, b) hard70, c) hardness with two classes, and d) geomorphic features.

The predictions of the most accurate models for hard90 and hard70 were illustrated in Fig 5 using a portion of study area (A1) to visually compare the predictions of the RF predictive models. This area was chosen as an example as it contains highly contrasting geomorphic features. For this particular portion, the match rate is 78.07% (i.e. with a corresponding mismatch rate of 21.93%, Table 11). Of the mismatched predictions, about 5.24% of hard predictions for hard70 were predicted as hard-soft for hard90, while about 13.83% of soft predictions for hard70 were predicted as hard-soft and soft-hard for hard90, resulting in low match rates for some classes between hard90 and hard70. Their predictions captured similar major patterns, while the some hard predictions for hard70 in the high banks were predicted as hard-soft or soft-hard for hard90 in the southern portion, and some soft predictions for hard70 on the terrace in the northeast corner were predicted as soft-hard and hard-soft for hard90. The hard substrates were found mostly on banks that were associated with the highest backscatter values (Fig 5D). The hard-soft and soft-hard substrates were also mostly found on banks as well as on portions of terraces. In comparison, soft substrates were mostly found on valleys that were often associated with the lowest backscatter values; and portions of terraces were also predicted as soft.

### Comparison of spatial predictions: four classes vs. two classes

The spatial predictions for hard90 and hard70 were similar with the predictions based on two hardness classes [7, 70]. The match rates were 92.06% and 93.18% respectively when the predictions of hard, hard-soft and soft-hard were pooled into one category (i.e. hard) for hard90 and hard70.

The spatial predictions for hard90 and hard70 in area A1 were similar with the predictions based on two hardness classes [7] (Fig 5), with the match rates of 81.53% and 89.42% respectively when the predictions of hard, hard-soft and soft-hard were combined into a single category (i.e. hard) for hard90 and hard70.

## Discussion and Conclusions

### Predictive accuracy of seabed hardness

All the models developed produced a good to perfect fit to the data (Tables 3 and 6); and their PA is also high (Figs 2 and 3). There is no definite relationship between the models’ fit and their PA, which is consistent with the findings in our previous study [7]. The PA of the most accurate models based on various FS techniques varies from 0.4852 to 0.6753 for hard90 and from 0.4116 to 0.5328 for hard70 in terms of *kappa* (Tables 9 and 10). According to Fielding & Bell [65], the agreement between the predicted and the tested values is good if *kappa* is between 0.4 and 0.75. This demonstrates that the PA of the models developed for predicting the seabed hardness is high. The PA of the most accurate models is even more notable in terms of *ccr*; and it varies from 84.62% to 89.78% for hard90 and from 84.09% to 87.04% for hard70. The high PA implies that: 1) seabed substrate was properly classified based on underwater video footage, and the data produced for the response variable is of high quality; 2) the hardness classification schemes developed (i.e. hard90 and hard70) are robust; and 3) the predictors used were informative. Furthermore, the PA for each model is stable and reliable because it is an averaged PA based on 100 repetitions of 10-fold cross-validation. Hence we can confirm that:

- sample size used in this study was adequate for modelling the seabed hardness;
- RF is an effective modelling method with high PA not only for presence/absence data but also for multi-level categorical data;
- robust predictive models were developed;
- seabed hardness of four classes can be predicted with a high accuracy; and
- RF can be applied to ‘small *p* and large *n*’ problems in environmental sciences, with the number of predictive variables as low as only one.

This study further affirms the superior performance of RF in marine environmental sciences [14, 15, 25, 26, 32]. The excellent performance of RF has been attributed to a number of features associated with RF including the ability to model non-linear relationships commonly found in the environmental sciences as discussed in previous studies [25, 26, 32].

It is apparent that non-soft classes were under-predicted while the soft class was over-predicted for both hard90 and hard70 data, which could be attributed to the unbalanced sample size of the hardness classes. The hard-soft class was under-predicted and approximately 50% of observed hard-soft samples were predicted as the soft class for both datasets, which was unexpected as it is anticipated to be more similar to hard or soft-hard classes than to the soft class. Although the limitations discussed in [7] could be potential factors resulting in such phenomenon, further studies are recommended to investigate this phenomenon. Approximately 50% of the non-soft classes were predicted as the soft class for hard70 data, which could be also attributed to the limitations discussed in [7]. This finding highlights the difference between hard90 and hard70, suggesting that hard90 data may have more accurately classified the seabed substrate and should be adopted in the future studies.

When the hard-soft and soft-hard classes were merged into the hard class, the user’s and producer’s accuracy between the observed and the predicted values was improved but still slightly lower than the previously published findings, particularly for hard70 data [7, 12]. This reduction in the accuracy could be attributed to that bathymetry was an important predictor in the previous studies [7, 12], but it is no longer an important predictor in this study. This change is because banks and terraces are mostly located in shallow water and are most often associated with hard substrates. However, the hard class in the previous study was split into three classes in the current study, which are located at similar water depths, thus bathymetry can no longer differentiate these classes and was not found to be an important predictor in this study.

### Predictive accuracy and correlated predictive variables

The PA changes with highly correlated predictors for models developed for hard90 and hard70 data. Their influence on the PA changes with individual predictors. The phenomenon that the inclusion of highly correlated predictors (i.e. *ρ*≥0.99 and *r*≥0.95) could improve the PA was observed for many models in this study (e.g. model 1 vs. model 43 and model 17 vs. model 19 for hard90, model 1 vs. model 26 for hard70, and those models compared in Table 10). This could be explained by the fact that these correlated predictors are informative as they have high variable importance (S3 File) and their inclusion can increase the number of informative predictors selected for each individual tree in RF, thus improving the PA. It was observed that including some correlated variables improved the PA in previous studies [7, 15, 34], suggesting that correlated variables may be able to compensate for the small number of predictors in environmental sciences, and more often than not we only have correlated proxy predictive variables in environmental sciences instead of causal predictors as seen in simulation studies [31].

In contrast, the exclusion of some highly correlated predictors may improve the PA in some other cases. It was observed for hard70 where bs27 and bs25 were highly correlated (*ρ* = 0.99, *r* = 1.00), but the exclusion of bs27 from model 4 led to a slight increase in PA for the subsequent model (i.e. model 5) containing bs25. The exclusion of bs25 from model 6 for hard70 also resulted in slight improvement in PA for model 7 that contains bs21. This phenomenon was further evidenced in Fig 2C after removing bs27 that was highly correlated with bs28 and bs33 (with *ρ*≥0.99 and *r* = 0.98). Similar findings were observed in previous studies [25, 32, 33, 35].

Contradictory findings were observed in previous studies in the marine environmental sciences as well as in this study regarding correlated predictors for RF. These opposite effects imply that not all highly correlated predictors should be used even if there are of high VI or excluded, and that there are no short-cuts in identifying the optimal predictive model. The *extendedForest* [39] package can efficiently deal with the correlated variables in terms of the variable importance, but selecting predictors that can improve PA from correlated predictive variables is a challenging task. No free lunch theorems [71] still apply even if the predictors are highly correlated. This finding also suggests that the typical approach used in pre-selecting predictors by excluding correlated variables (i.e. *r*≥0.95 or the inflation factor ≥20) needs to be re-examined for identifying predictive models using machine learning methods, at least for the application of random forest in marine environmental sciences. These applications further demonstrate that feature selection is essential for identifying an optimal predictive model for RF in marine environmental sciences [15, 25].

### Predictive accuracy and important and unimportant predictors

Some important and unimportant predictors were identified based on the VI for hard90 and the AVI for hard70. They were excluded during the initial FS process, which may obviously lead to the exclusion of some important predictors and thus result in less accurate predictive models which has also been observed in previous studies [7, 64]. Adding the important predictors and removing the unimportant ones could partially solve this problem. However, the accuracy of predictive models in this study showed inconsistent response patterns to the inclusion or removal of the important or unimportant predictors. Of the three important predictors identified for hard90, two of them (i.e. variance and surface) further improved the PA after adding them back while the inclusion of the remaining one (i.e. relief) reduced the PA (Table 3 and Fig 2A). Of the two identified important variables for hard70, the inclusion of bs21 further improved the PA while the inclusion of bathy.moran reduced the PA; and of the three unimportant variables, removing profile.curv and bs.moran further improved the PA, whereas removing variance reduced the PA (Table 6 and Fig 3A). It is apparent that their influence on PA changes with individual predictors. It was also observed that reliance upon variable importance only can lead to suboptimal predictive model and the most accurate predictive model may be overlooked in previous studies [7, 64]. These findings suggest that the predictor(s) with the least variable importance should not be excluded without further testing. The detection of the important and unimportant predictors provides signals of potential candidates or establishes prior information for further improvement of the PA. However efforts are required to select relevant predictors from them to further improve the PA.

### Feature selection methods

Five FS methods, VI, AVI, KIAVI, Boruta and RRF, were tested in terms of PA in this study. They are essentially all VI-based wrapper methods according to Janecek *et al*. and Saeys *et al*.[28, 62]. These methods were applied to the full dataset and a pre-selected sub-dataset. Since the VI was obtained from RF, these methods are also embedded techniques [28]. To develop the final predictive models based on VI and AVI, the contribution to the PA of relevant predictors was used to determine their inclusion or elimination in the backward or forward stepwise selection. Hence, these applications cover three different FS techniques: filter, wrapper and embedded. In this study, filter and/or wrapper FS techniques were used in combination with embedded FS techniques within RF.

It is apparent that all FS techniques used in this study improved the PA except that the filter FS method significantly reduced the PA with respect to the full models. The effects of these techniques are however different for hard90 and hard70. For hard90, the model developed by applying AVI to 41 variables is significantly more accurate than other FS techniques. For hard70, the model developed by applying Boruta to 41 variables is significantly more accurate than other FS techniques. These findings highlight that: 1) these FS techniques are data sensitive and data-specific, and 2) to obtain an optimal predictive model for a given dataset, relevant FS techniques should be tested to select the most appropriate FS technique, otherwise sub-optimal models may be produced. All the most accurate models identified in Tables 9 and 10 are believed to be the local optimum, which were identified under the FS methods with the limited resources used. In fact, all optimal models identified based on FS methods are believed to be local optimal. A complete search for the global optimal model(s) is time consuming and at times, impossible, especially when there are a large number of predictive variables. This is because the computational requirements have a factorial increase with the number of variables, highlighting the importance of FS methods. Although the application of KIAVI did not further improved the PA, it was found that informing knowledge to VI can further improve the PA [7, 64]. Again no free lunch theorems [71] are still effective when using FS methods. In general, AVI and Boruta show their effectiveness in searching for the most accurate predictive models and are recommended for future studies. It may be worth testing whether applying the AVI to the variables selected by Boruta could further improve the PA in future studies. However, caution should be taken when applying the filter FS method because: 1) the number of predictive variables is usually small in environmental sciences; and 2) inclusion of highly correlated predictors (with r ≥ 0.99) could improve the PA as discussed above.

Computational demand is critical in choosing a FS method. Among the five methods, the computational time decreases from VI, AVI, KIAVI, Boruta to RRF in terms of the number of models tested to find the final model. Three advantages of VI were discussed previously [34]. KIAVI and AVI share these advantages and AVI led to the most accurate model for hard90 in this study, but apparently their computational demand is high and automated programs need to be developed to increase their computational efficiency in the future. Moreover, these FS methods are applicable to datasets with small number of features such as those tested in this study. For datasets with large number of features (e.g. from thousands to millions), dimension reduction methods may be required to reduce the computing demand. However, caution needs to be taken when non-linear correlations exist [72].

As discussed in the previous study [7], the PA is the ultimate measure for selecting the predictive model. Model selection via these FS methods is based on the PA. These FS methods will produce models that are the most accurate or optimal instead of the most parsimonious as discussed above and previously [7]. The traditional model selection methods such as AIC and BIC for regression models (e.g. linear model, generalised linear model) attempt to select the most parsimonious models that are not necessarily the most accurate models, especially when proxy variables are used as predictors instead of causal variables. Since the ultimate goal of predictive modelling is to identify the most accurate model(s), these FS methods are more appropriate than AIC and BIC methods in selecting predictive model(s). The principles underpinning these FS methods can be easily applied to other machine learning methods as well as regression models. Therefore, they are recommended for selecting predictive model(s) for RF and other modelling techniques in future studies.

### Hardness classification methods and prediction maps of seabed hardness

#### Hard90 vs hard70

Two seabed hardness classification schemes, hard90 and hard70, were proposed in this study. In comparison with hard70, the advantages of Hard90 were presumed to be that: 1) the probabilities of ‘hard’ and ‘soft’ are increased accordingly by using a threshold of 90% or 10%, which ensured the ‘hard’ or ‘soft’ substratum classification to be composed mostly of the representative substratum (i.e. ‘hard’ or ‘soft’); and 2) number of samples for the mixed classes of hardness are increased and samples are more evenly distributed among four classes. Thus the PA was expected to be higher for models based on hard90 than on hard70, which were confirmed by the findings in this study (Tables 9 and 10).

Less hard and soft and more mixed classes were also anticipated to be predicted for hard90 than for hard70, which are confirmed by the findings in Table 11. Hard predictions for hard70 predicted as the hard-soft class for hard90 was less than soft predictions for hard70 predicted as the soft-hard class for hard90 (1.26% vs. 3.12%); even a portion of soft predictions for hard70 (2.06%) was predicted as hard-soft for hard90. However, the majority of the predictions are similar for hard90 and hard70, with a match rate as high as 92.31% between these two schemes (Table 11). By comparing the distribution of the hardness classes in the hard70 and hard90, there were three samples that shifted from hard to hard-soft, and from soft to soft-hard respectively, but the resultant changes in predictions are not as even as the samples.

#### Four classes vs. two classes

The PA for four hardness classes in this study seems less than that for two classes in the previous study [7]. A few factors were expected to affect the PA either positively or negatively:

1. The hard class in the previous study was further divided into three classes in this study, so the PA was expected to be reduced.
2. Since the locations of the video tracks are again different to their true locations (thus to the locations of backscatter data), we should not expect to reduce the predictive error much by using the predictive variables at video locations directly.
3. The predictive variables derived in this study are expected to be more reliable than those in previous studies [7, 12], because in previous studies the variables were derived for the sample locations that are usually a few meters to a few kilometres away from the video track, while in this study all variables were generated for the location of the video locations to remove the possible effects of the inaccuracy in the variables caused by the distance. Hence an accuracy improved model is expected.
4. Bathymetry was an important predictor for hard and soft in the previous study [7] but not in the current study as discussed above in 4.1. This may further explain why the PA was slightly reduced in this study.

The spatial predictions for hard90 and hard70 were similar with the predictions based on two hardness classes [7], with match rates as high 92.06% and 93.18% respectively. They are also as high as 81.53% and 89.42% respectively for A1. These findings show that major patterns were captured in their predictions, although predictions based on hard70 as expected are more similar to the predictions based on two classes than the predictions based on hard90. This is because both the two hardness class system in the previous study [7] and hard70 scheme in this study used the same 70% and 30% thresholds to classify hard and soft classes.

#### Geomorphic features

The predicted maps reflect the influence of various geomorphic features such as banks, terraces, and valleys (Figs 1C and 5). The associations of the predicted hardness with geomorphic features are generally similar to those discussed in previous studies [7, 12]. These associations were supported by ecological studies because certain organisms expected to be found on hard [43, 73] or soft substrates [43, 73] were observed in the corresponding substrates in this study.

The predictions that were illustrated using a portion of study area (A1) (Fig 5) highlight the difference in predictions among all geomorphic features for hard90 and hard70, with a lower match rate than that for all areas (78.07% vs 92.31%) and with more mixed classes predicted for hard90 (Table 11). Such differences were mainly observed on banks and terraces (Figs 1C and 5).

### Limitations and other issues

The nature of the seabed is a fundamental factor in controlling the acoustic returns (i.e. backscatter). Soft seabed substrates generally produce lower backscatter intensity; in contrast, hard seabed substrates generally produce higher backscatter intensity [74–77]. However, the strength of the acoustic returns can be significantly attenuated or even lost because of scattering from surface topography and the sessile benthic organisms, causing an apparent reduction in the backscatter intensity. Likewise, very strong seabed reflections can be observed due to well-sorted unconsolidated sediments in the absence of any surface topography or the presence of hard surface underneath a veneer of soft materials. The existence of these factors can affect the reliability of acoustic data and classification of video images [12], thus affecting the PA.

In addition, the limitations discussed in relation to using video footage to validate substrate grounds and to the nature of backscatter intensity in Li *et al*. [7] are also applicable to this study.

## Conclusions

Two seabed hardness classification schemes proposed in this study, hard90 and hard70, are effective and they all led to high PA for seabed hardness predictions. Seabed hardness is predictable and can be predicted into a spatially continuous layer with a high accuracy, especially to large areas where multibeam acoustic data exist and predictions of seabed classes are needed for marine planning and management.

Not all highly correlated predictive variables should be used or excluded. The usual approach used in pre-selecting variables by excluding correlated ones should be re-examined for identifying predictive models using machine learning methods, at least for the application of RF in the environmental sciences.

The identification of the important and unimportant predictors provides guideline for further improving the predictive models, although additional effort is required to select relevant predictors from the important and unimportant predictors.

FS is essential for identifying an optimal predictive model for RF in environmental sciences but is a challenging task as a complete search for the global optimal model(s) is time consuming and sometimes impossible. AVI and Boruta show their effectiveness in searching for the most accurate predictive models and are recommended for future studies. Automated computational programs for AVI are recommended to be developed to improve its computational efficiency in the future. However, caution should be taken when applying filter FS method in selecting optimal predictive models.

This study further affirms the superior performance of RF in marine environmental sciences. RF is an effective modelling method with high PA not only for presence/absence data and but also for multi-level categorical data. RF can be applied to ‘small *p* and large *n*’ problems in environmental sciences. It is recommended for generating spatially continuous predictions of categorical variables like seabed hardness when the information of relevant predictive variables is available.

## Supporting Information

S1 Fig The relationships between seabed hardness (i.e. total of hard) and 20 predictive variables. (DOCX)

S1 File The definitions and representative images of seven size-class categories of seabed substratum composition. (DOCX)

S2 File The dataset for developing predictive models in this paper.csv. (CSV)

S3 File Variable importance (VI) for hard90. Fig A: measured by RF using randomForest package for 20 predictive variables [1]; Fig B: averaged VI from 100 iterations of RF using extendedForest package for 20 variables [2]; and Fig C: averaged VI from 100 iterations of RF using extendedForest package for 41 variables [2].
(DOCX)
