# Supplementary figures and images for: Selecting Optimal Random Forest Predictive Models: A Case Study on Predicting the Spatial Distribution of Seabed Hardness
Source: PLoS One. 2016 Feb 18;11(2):e0149089. doi: 10.1371/journal.pone.0149089 (PMC4758710; doi:10.1371/journal.pone.0149089)

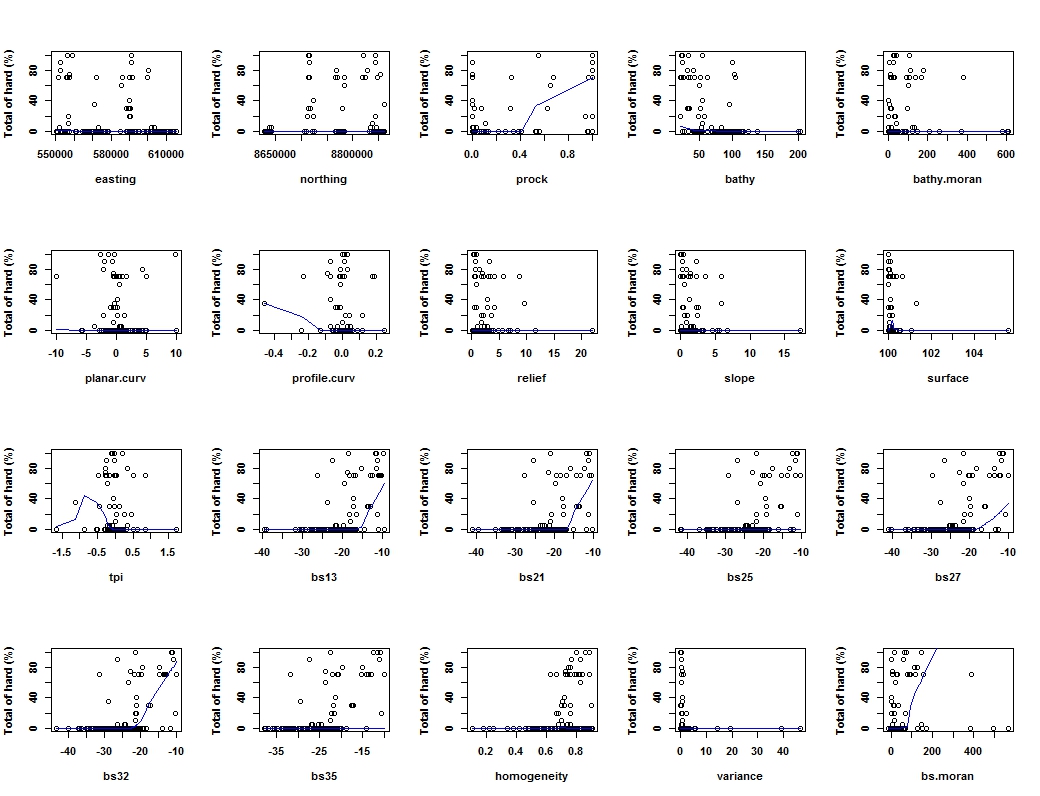


**S3 Fig.** The relationships between seabed hardness (i.e. total of hard) and 20 predictive variables.

Supplement: S1 Fig — (DOCX) [file pone.0149089.s001.docx]
