# Supplementary material for: Selecting Optimal Random Forest Predictive Models: A Case Study on Predicting the Spatial Distribution of Seabed Hardness
Source: PLoS One. 2016 Feb 18;11(2):e0149089. doi: 10.1371/journal.pone.0149089 (PMC4758710; doi:10.1371/journal.pone.0149089)
Supplement: S1 File — (DOCX) [file pone.0149089.s002.docx]

**S1 File:** The definitions and representative images of seven size-class categories of seabed substratum composition

This section comprises an image library of representative substratum types using underwater imagery from Geoscience Australia marine surveys SOL5117 and SOL4934 of the Joseph Bonaparte Gulf. We followed a similar size-class characterization scheme outlined by Wentworth (1922) and Stein, Tissot et al. (1992) and Mortensen & Buhl-Mortensen (2005), which separates substratum into seven clearly defined size-class categories:

1) Bedrock (Appendix A1),

2) boulders (≥ 25.5 cm) (Appendix A2),

3) cobbles (≥ 6.5 to < 25.5 cm) (Appendix A3),

4) pebble/rubble (≥ 4mm to < 6.5 cm) (Appendix A4),

5) gravel (≥ 2 to < 4 mm) (Appendix A5),

6) sand (≥ 63 µm to < 2mm) (Appendix A6) and

7) mud (< 63 µm) (Appendix A7).

| 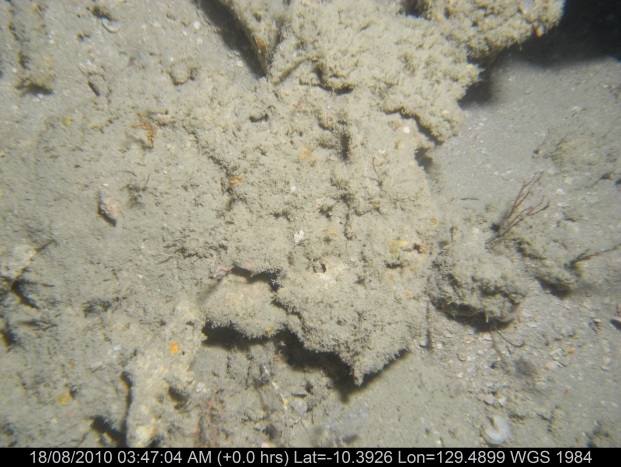 | 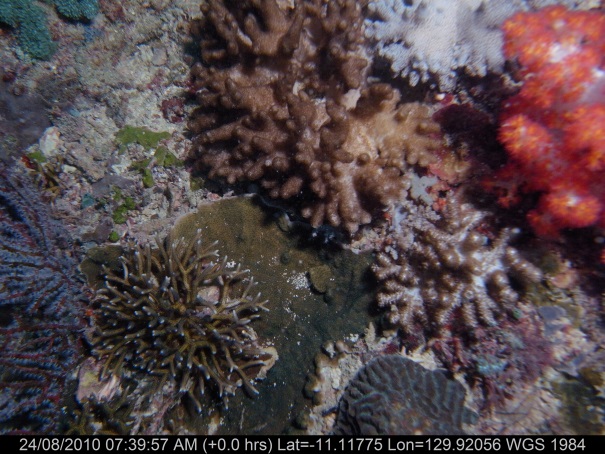 |
| --- | --- |
| 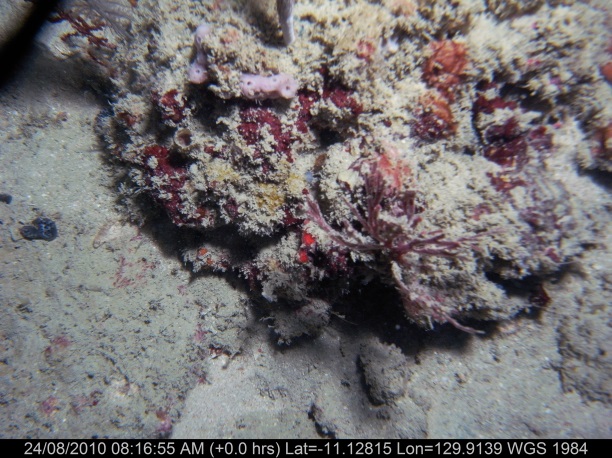 | 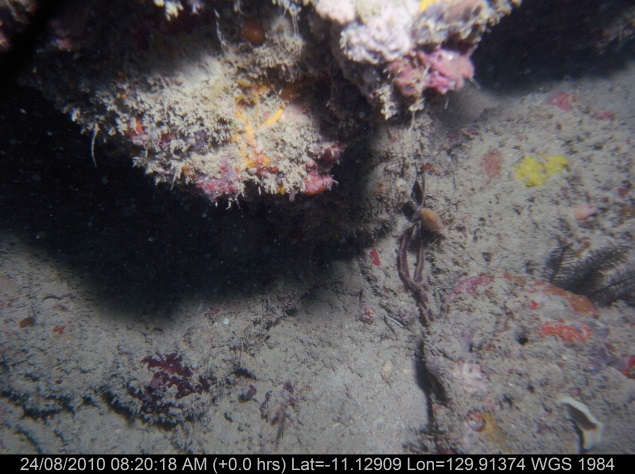 |

S1.1. Representative images of bedrock substratum category. These are areas of consolidated hard material, comprising moderate to high relief and are often found with various growth forms of sessile animals (e.g. octocorals, soft corals, corals). a) *stn48cam49 (SOL5117)* rocky outcrops with hydroids and gorgonians with thin veneer of sediment (80% bedrock in figure); b) *stn72cam73 (SOL5117)* branching and encrusting hard coral, soft coral and octocoral forms on bedrock (95% bedrock); c) *stn73cam74 (SOL5117)* encrusting and branching red algae on rocky outcrops; (80% bedrock) and d) *stn73can74 (SOL5117)* red algae and encrusting sponge on rocky outcrops (85% bedrock)*.*

| 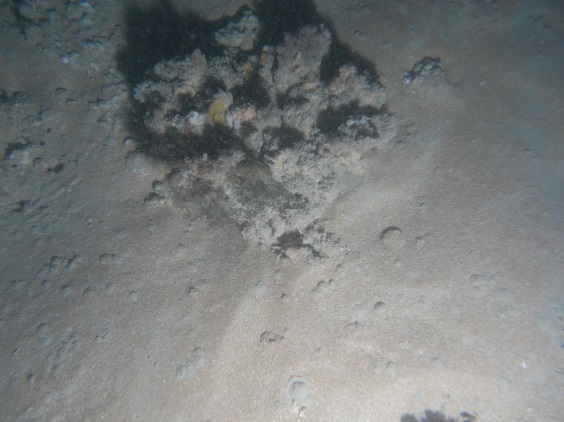 | 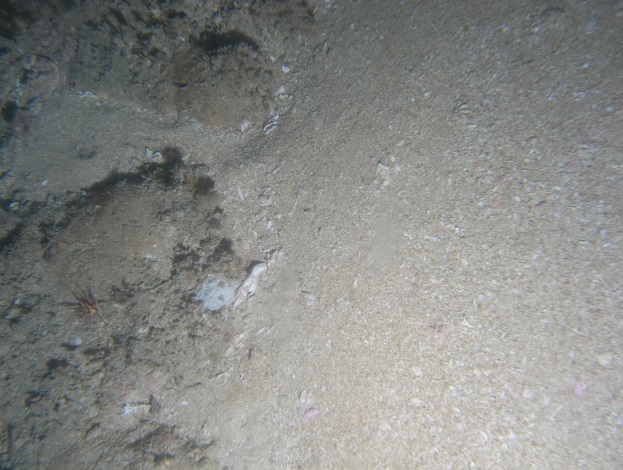 |
| --- | --- |
| 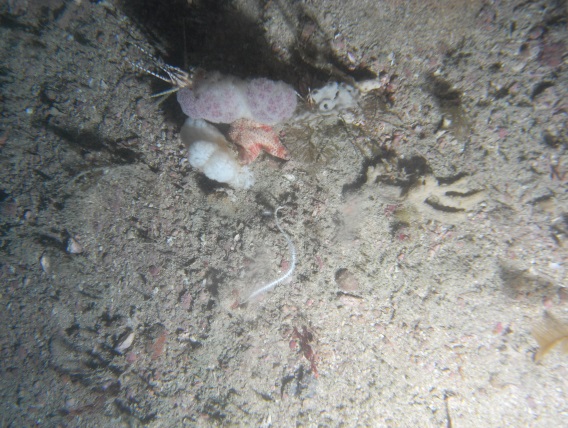 | 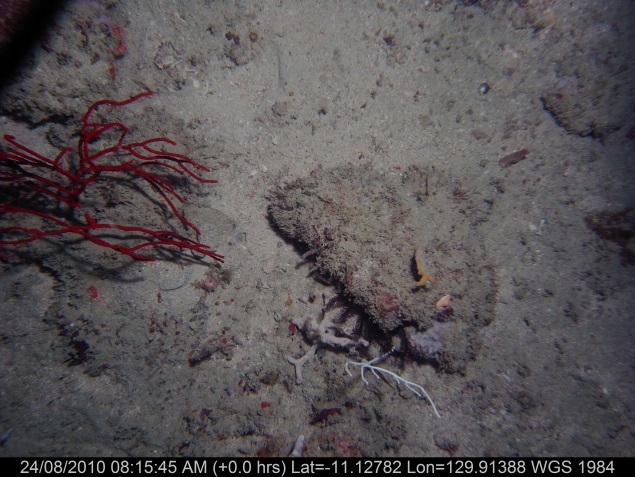 |

S1.2. Representative images from the boulder substratum category. Boulders are often distinctive in shape and isolated from areas of bedrock. Boulders can also be covered with a thin veneer of sediment and growth of sessile fauna, occasionally with evidence of rolling (absence of sessile fauna). a) *stn30cam18 (SOL4934)* boulders in areas of sandy sediments (25% boulder with 75% soft substratum); b) *stn33cam23(SOL4934)* boulders creating depression in localised areas (30% boulders with 70% soft substratum); c) *stn33cam23 (SOL4934)* boulder with sessile fauna (octocorals *Dendronepthea sp*, sponges and hydroids) and often attracting mobile epibenthic fauna (60% boulders as evidenced by amount of epifauna); and d) *stn73cam74(SOL5117)*  boulder field with evidence of sessile growth (60% boulders with 40% soft substratum)*.*

| 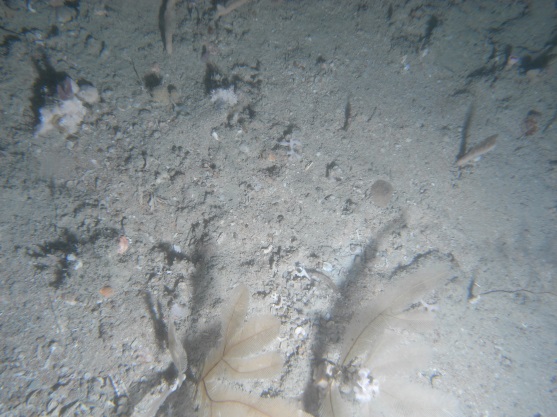 | 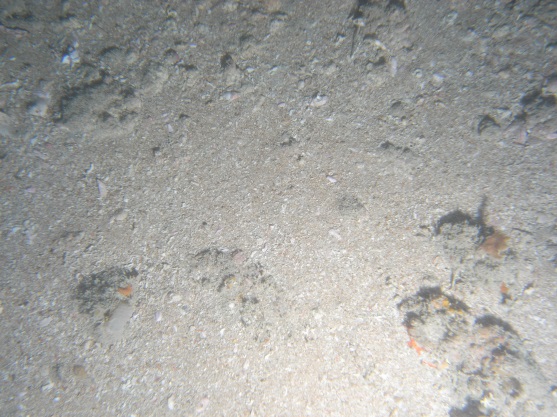 |
| --- | --- |
| 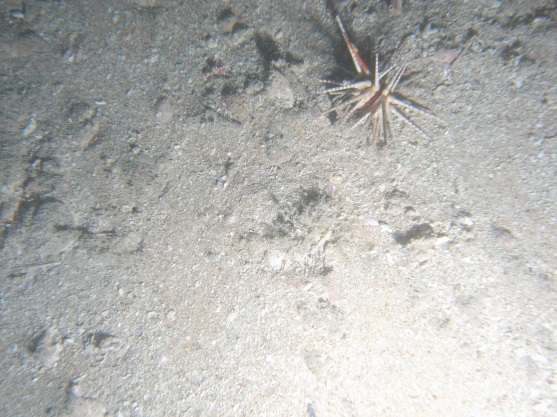 | 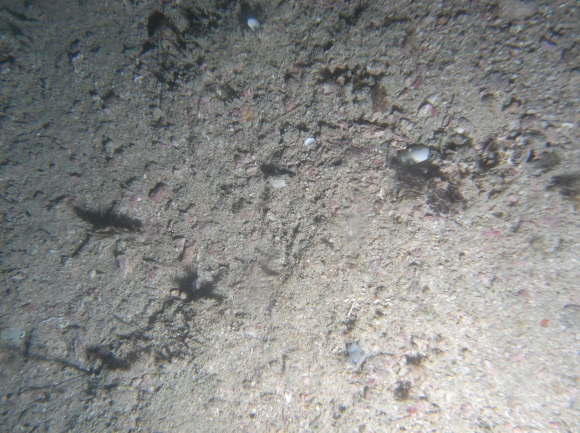 |

S1.3. Representative images from the cobble substratum category. This substratum type may be covered with a fine veneer of sand/mud as seen in our examples of the Joseph Bonaparte Gulf and occur with sessile epifaunal biota not normally associated with sand-mud habitats. a) *stn02cam20 (SOL4934)* cobbles covered with thin veneer of sand/mud and associated sessile fauna (hydroids and sponges) (30% cobbles with 70% soft substratum); b) *stn31cam19* *(SOL4934)* cobbles covered with thin veneer of sediments and encrusting sp. of sponge (30% cobbles with 70% soft substratum); c) *stn31cam19* *(SOL4934)* cobbles with thin veneer of mud/sand (20% cobbles with 80% soft substratum); and d) *stn33cam23 (SOL4934)* cobbles with thin veneer of sand and gravel (30% cobbles with 70% soft substratum).

| 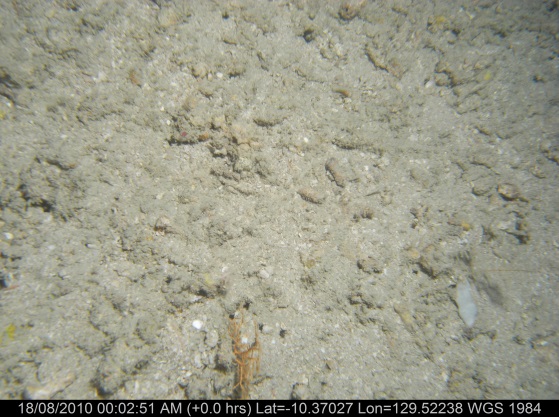 | 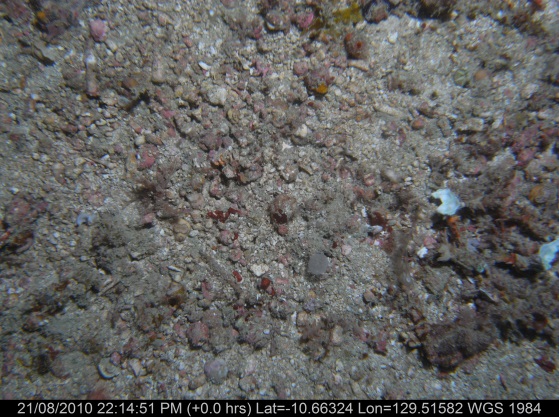 |
| --- | --- |
| 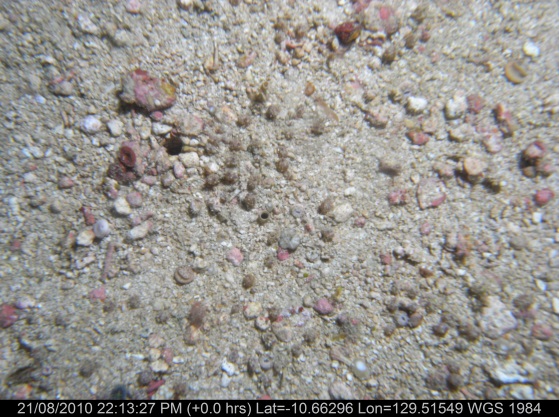 | 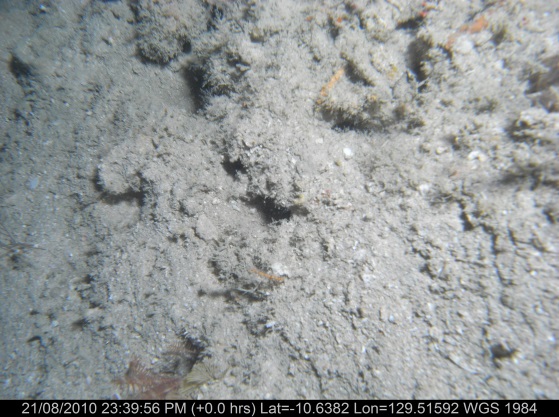 |

S1.4. Representative images from the rubble/pebble category. Rubbles and pebbles may show evidence of rolling. In addition, encrusting algae is commonly present and may promote growth of other sessile benthic animals. a) *stn46cam47 (SOL5117)* fine sediment veneer with evidence of sessile fauna (60% rubbles/pebbles and 40% soft substratum); b) *stn56cam57 (SOL5117)* encrusting red algae on rubble (70% rubbles/pebbles and 30% soft substratum); c) *stn56cam57 (SOL5117)*  rubble associated with encrusting red algae and solitary hard corals (50% rubbles/pebbles and 50% soft substratum); and d) *stn57cam58 (SOL5117)* fine sediment veneer on rubble (40% rubbles/pebbles and 60% soft substratum).

| 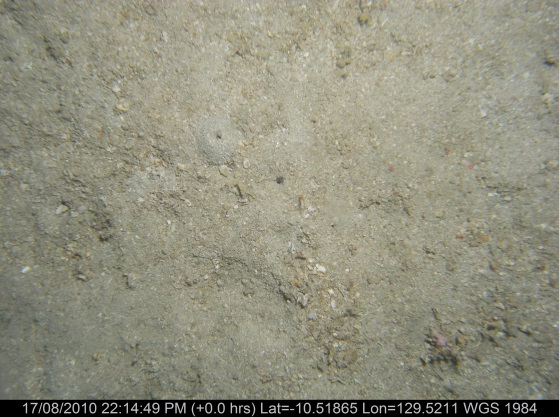 | 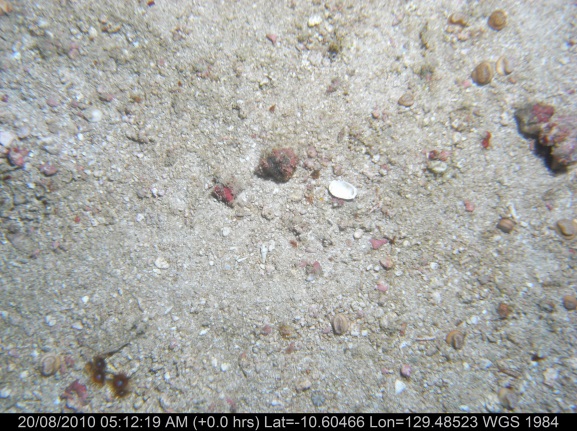 |
| --- | --- |
| 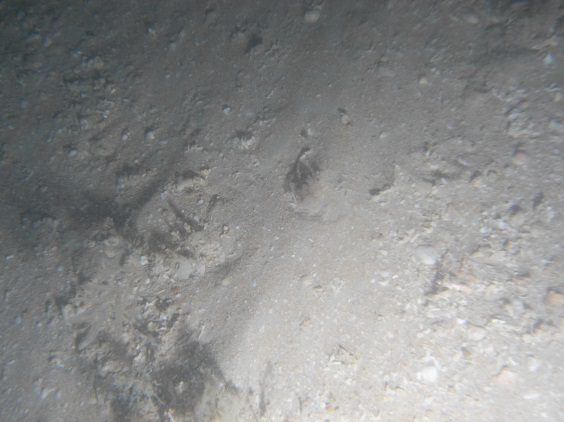 | 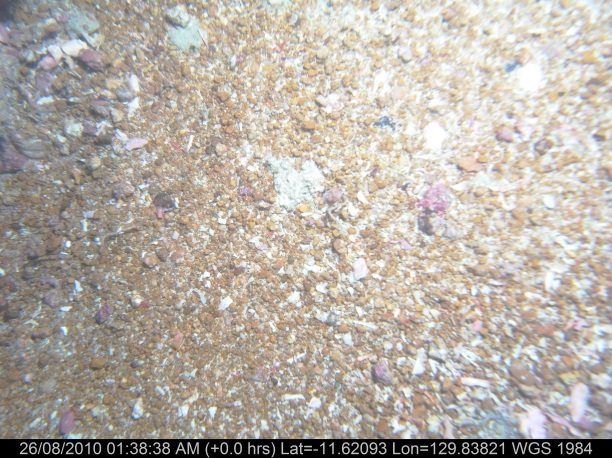 |

S1.5. Representative images of gravel. Gravel can often contain shelly sediments in conjunction with sandy/muddy sediments. a) *stn45cam46 (SOL5117)* gravel interspersed with sandy and shelly sediments (40% gravel and 60% mud/sand); b) *stn55cam56 (SOL5117)* gravel interspersed with sandy and shelly sediments and some solitary hard corals (70% gravel and 30% mud/sand); c) *stn63cam45 (SOL4934)* gravel interspersed with sandy and shelly sediments (35% gravel and 65% sand/mud); and d) *stn81cam77 (SOL5117)* gravelly sediments with some encrusting red algae (100% gravel).

| 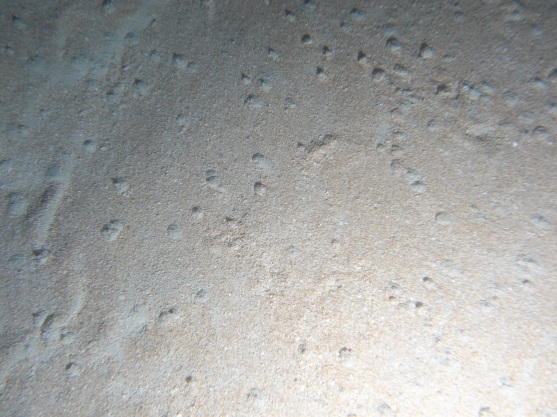 | 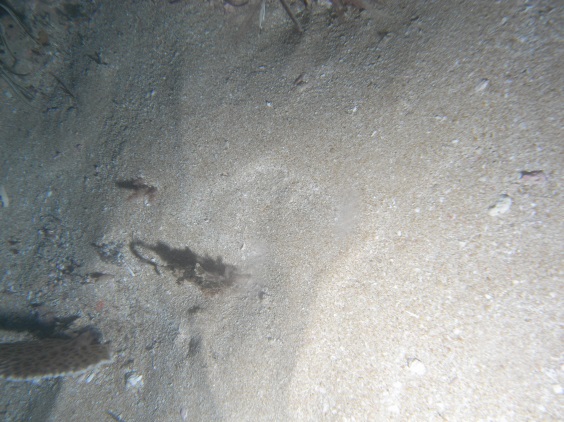 |
| --- | --- |
| 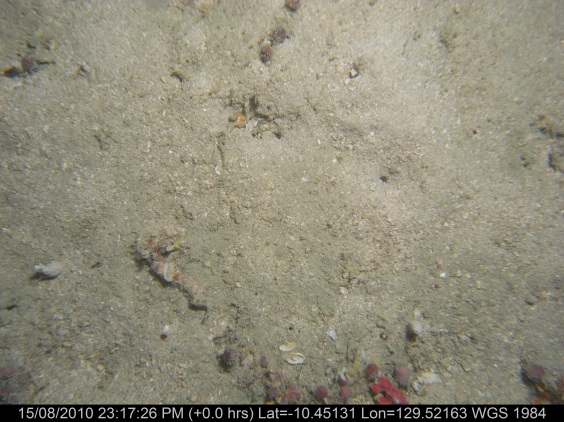 | 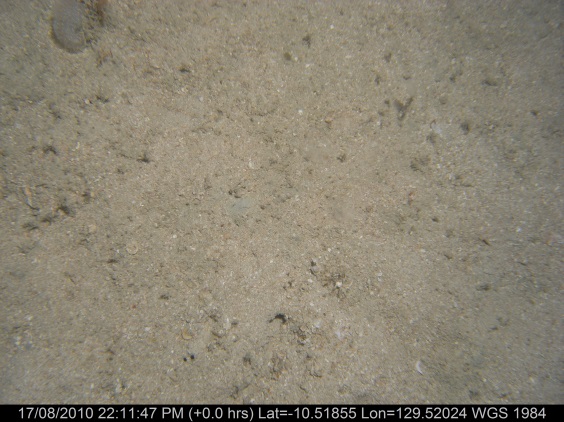 |

S1.6. Representative images of sand. Sand was not always possible to distinguish between mud, and often associated with bioturbation from infaunal animals. a) *stn30cam18 (SOL4934)* sandy sediments (100% sand); b) *stn33cam23 (SOL4934)* sandy sediments with evidence of slightly rippled relief (80% sand, 15% boulder 5% gravel); c) *stn44cam42 (SOL5117)* sandy sediments with evidence of bioturbation (burrows) (80% sand, 10% gravel, 10% cobbles); and, d) *stn45cam46 (SOL5117)* sandy sediments with evidence of bioturbation (burrows) (90% sand and 10% gravel).

| 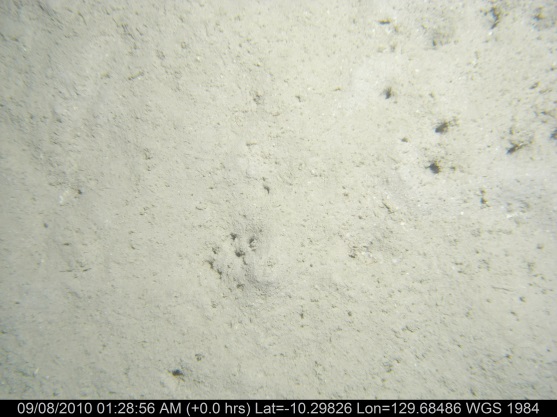 | 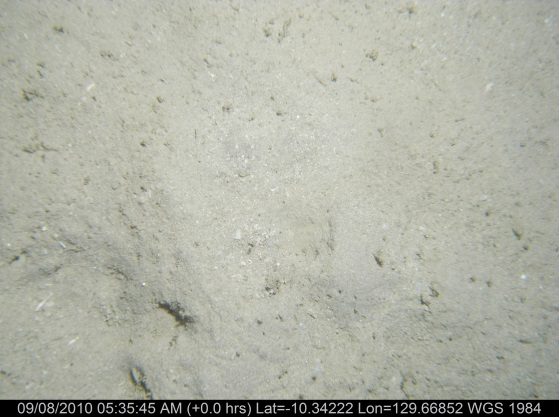 |
| --- | --- |
| 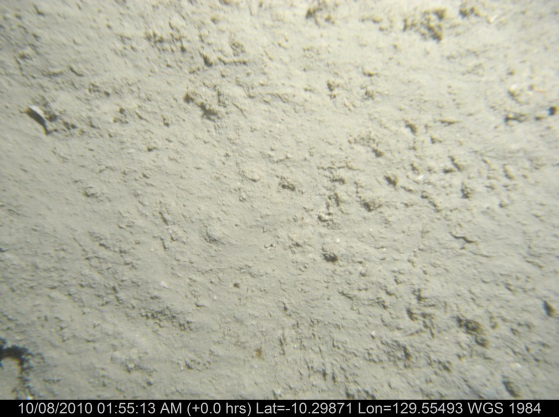 | 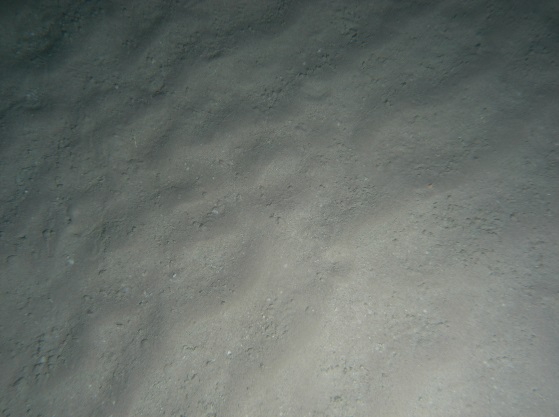 |

S1.7. Representative images of mud. As mentioned, it was not always possible to distinguish the difference between mud and sand. In addition, muddy sediments, much like sand, was often associated with bioturbation tracks and marks of craters, pits, mounds and burrows. a) *stn29cam30 (SOL5117)* muddy sediments with evidence of bioturbation (pits) (100% mud); b) *stn31cam32 (SOL5117)* muddy sediments with evidence of bioturbation (burrows) (95% mud and 5% gravel); c) *stn32cam33 (SOL5117)* muddy sediments with evidence of bioturbation (95% mud and 5% gravel); d) *stn38cam26 (SOL4934)* rippled muddy sediments (100% mud).

**Reference**

Wentworth C.K. (1922). A scale of grade and class terms for clastic sediments. Journal of Geology 30, 377-392.
