# Supplementary material for: Selecting Optimal Random Forest Predictive Models: A Case Study on Predicting the Spatial Distribution of Seabed Hardness
Source: PLoS One. 2016 Feb 18;11(2):e0149089. doi: 10.1371/journal.pone.0149089 (PMC4758710; doi:10.1371/journal.pone.0149089)
Supplement: S3 File — Fig A: measured by RF using randomForest package for 20 predictive variables [1]; Fig B: averaged VI from 100 iterations of RF using extendedForest package for 20 variables [2]; and Fig C: averaged VI from 100 iterations of RF using extendedForest package for 41 variables [2]. (DOCX) [file pone.0149089.s004.docx]

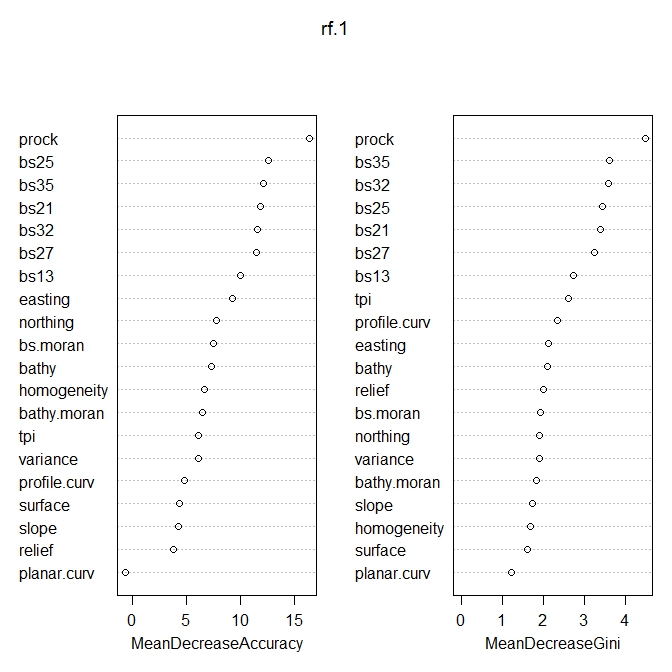

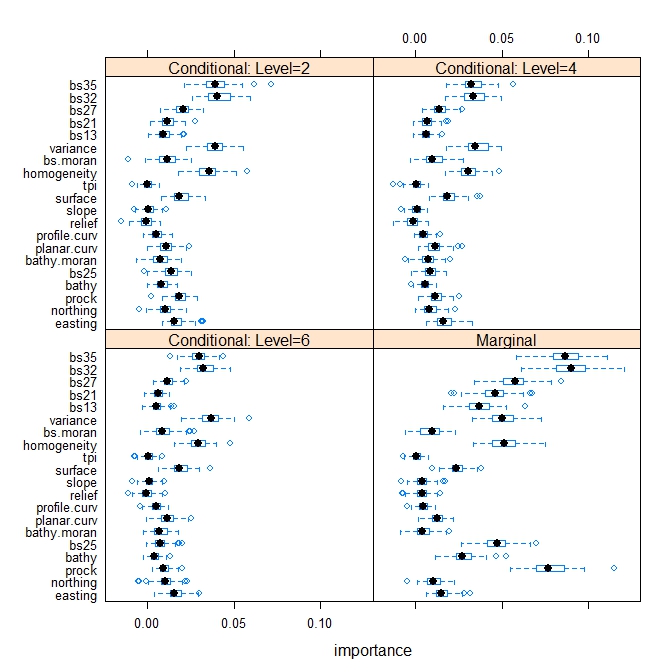


**Fig A**

**Fig B**

**
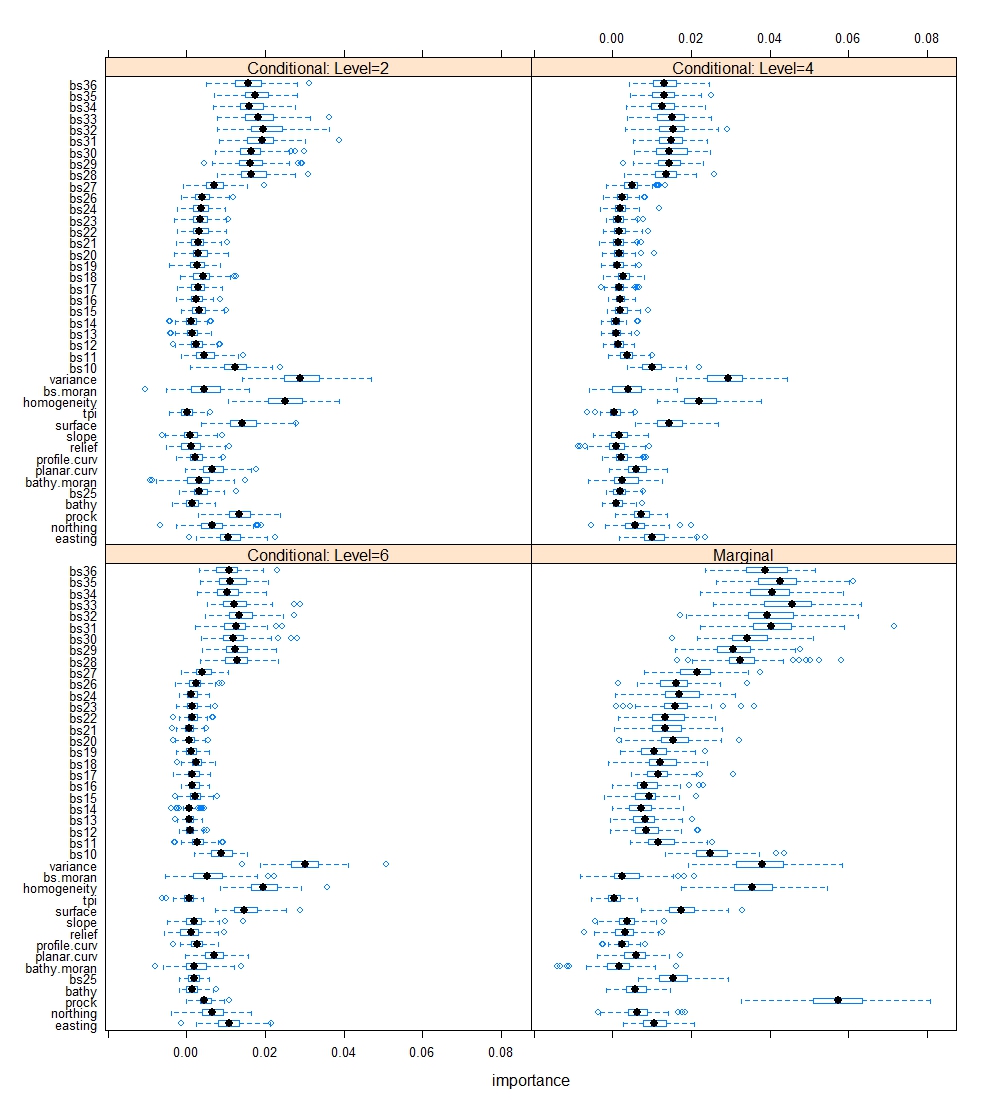
**

**Fig C**

**S3 File.** Variable importance (VI) for hard90. Fig A: measured by RF using randomForest package for 20 predictive variables [[1](#_ENREF_1)]; Fig B: averaged VI from 100 iterations of RF using extendedForest package for 20 variables [[2](#_ENREF_2)]; and Fig C: averaged VI from 100 iterations of RF using extendedForest package for 41 variables [[2](#_ENREF_2)].

1. Liaw A, Wiener M. Classification and regression by randomForest. R News. 2002;2(3):18-22.

2. Smith SJ, Ellis N, Pitcher CR. Conditional variable importance in R package extendedForest. R vignette 〈<http://gradientforestr-forger-projectorg/Conditional-importancepdf〉>. 2011.
